# Mib1 prevents Notch *Cis*-inhibition to defer differentiation and preserve neuroepithelial integrity during neural delamination

**DOI:** 10.1371/journal.pbio.2004162

**Published:** 2018-04-30

**Authors:** Chooyoung Baek, Lucy Freem, Rosette Goïame, Helen Sang, Xavier Morin, Samuel Tozer

**Affiliations:** 1 Cell Division and Neurogenesis, Institut de Biologie de l’Ecole Normale Supérieure (IBENS), Ecole Normale Supérieure, CNRS, Inserm, PSL Université Paris, Paris, France; 2 Sorbonne Universités, UPMC Univ Paris 06, IFD, Paris, France; 3 The Roslin Institute and Royal (Dick) School of Veterinary Studies, The University of Edinburgh, Easter Bush, Midlothian, United Kingdom; 4 Sorbonne Universités, UPMC Univ Paris 06, UMRS 968, UMR 7210, Institut de la Vision, Paris, France; California Institute of Technology, United States of America

## Abstract

The vertebrate neuroepithelium is composed of elongated progenitors whose reciprocal attachments ensure the continuity of the ventricular wall. As progenitors commit to differentiation, they translocate their nucleus basally and eventually withdraw their apical endfoot from the ventricular surface. However, the mechanisms allowing this delamination process to take place while preserving the integrity of the neuroepithelial tissue are still unclear. Here, we show that Notch signaling, which is classically associated with an undifferentiated state, remains active in prospective neurons until they delaminate. During this transition period, prospective neurons rapidly reduce their apical surface and only later down-regulate N-Cadherin levels. Upon Notch blockade, nascent neurons disassemble their junctions but fail to reduce their apical surface. This disrupted sequence weakens the junctional network and eventually leads to breaches in the ventricular wall. We also provide evidence that the Notch ligand Delta-like 1 (Dll1) promotes differentiation by reducing Notch signaling through a *Cis*-inhibition mechanism. However, during the delamination process, the ubiquitin ligase Mindbomb1 (Mib1) transiently blocks this *Cis*-inhibition and sustains Notch activity to defer differentiation. We propose that the fine-tuned balance between Notch *Trans*-activation and *Cis*-inhibition allows neuroepithelial cells to seamlessly delaminate from the ventricular wall as they commit to differentiation.

## Author summary

The process of neural delamination, whereby nascent neurons detach from the ventricular surface of the neural tube after differentiation, is still poorly characterized. The vertebrate neural tube is initially exclusively composed of neuroepithelial progenitors whose apical attachments ensure the integrity of the ventricular wall. However, as differentiation takes place, increasing numbers of progenitors exit the cell cycle and delaminate, therefore challenging the integrity of the apical surface. Here, we have analyzed the mechanisms underlying the delamination process in the neuroepithelial tissue. We show that the Notch signaling pathway is active in all progenitors and that its repression is critical for prospective neurons to commit to differentiation. Moreover, we find that the Notch ligand Delta-like 1 (Dll1) represses Notch activity through *Cis*-inhibition of the Notch receptor and induces differentiation. Strikingly, we show that the ubiquitin ligase Mindbomb1 blocks the *Cis*-inhibition process and allows Notch activity to be transiently sustained, which defers differentiation. This transition period is essential for prospective neurons to constrict their apical domain before delamination, as the alteration of this sequence results in breaches in the ventricular wall, followed by massive tissue disorganization. Taken together, our results reveal that the temporal control of Notch down-regulation needs to be tightly coordinated with the delamination process to preserve the integrity of the ventricular wall while allowing neuroepithelial cells to differentiate.

## Introduction

The vertebrate neuroepithelium is initially composed of elongated progenitors polarized along the apical–basal axis that actively proliferate. After a phase of expansion, these progenitors start producing neurons through asymmetric and eventually symmetric neurogenic divisions. Following mitosis, daughter cells committed to differentiation translocate their nucleus to the basal side of the neural tube (NT) before they delaminate from the ventricular surface. Neuroepithelial cells are attached to their neighbors through apical junctional complexes. As they enter differentiation, they down-regulate N-Cadherin levels, a prerequisite for the retraction of the apical endfoot and expression of neuronal markers [1, 2]. Nevertheless, the cellular events that accompany the delamination process and make it compatible with the maintenance of tissue integrity are still unclear.

The balance between proliferation and differentiation in the NT, although involving a long list of regulators, relies at its core on the antagonistic action of Notch downstream targets and proneural genes [3]. Notch signaling plays a well-documented role in binary fate decisions in many systems and specifically promotes the maintenance of the undifferentiated state in the nervous system [4–7]. On the other hand, proneural genes are basic helix-loop-helix (bHLH) transcription factors that promote cell cycle exit and neural commitment [8]. Thus, neural differentiation is accompanied by increased levels of proneural gene expression and loss of Notch activity. However, the functional connection between these two processes during the transition from progenitor to neuron remains to be clarified. Although proneural genes induce differentiation, they cannot directly inhibit Notch signaling. Instead, they control the expression of Notch ligands [9–12], which were shown to promote differentiation in individual cells [13, 14]. However, their mode of action during that process has proven difficult to characterize. According to the "lateral inhibition with feedback" model, the increased expression of Notch ligands in the signal-sending future neuron would strongly “*Trans*”-activate Notch signaling and therefore down-regulate Notch ligand expression in the neighboring progenitors. These would, in return, poorly *Trans*-activate Notch in the signal-sending cell, and shutdown of the signaling pathway would allow this cell to differentiate [15]. While there is good evidence suggesting that increased Notch ligand expression inhibits differentiation non–cell autonomously (i.e., through lateral inhibition) [16, 17], whether a feedback mechanism down-regulates Notch activity in the signal-sending cell has not been proven in vertebrates. On the other hand, studies in *Drosophila* have shown that Notch ligands are able to inhibit the signaling activity of Notch receptors present in the same cell, a process termed “*Cis*”-inhibition [18–20]. This would in theory allow the direct inhibition of Notch receptors by their ligands in the differentiating cell. In vitro experiments and overexpression studies in vivo have shown that the ability of Delta ligands to *Cis*-inhibit Notch receptors is conserved in vertebrates [21, 22]. However, proving the existence of *Cis*-inhibition in vivo is hampered by the fact that Notch ligand loss-of-function will affect both *Trans*- and *Cis*- activities. In this regard, Delta-like 3 (Dll3) represents an interesting exception to the rule, as it can *Cis*-inhibit Notch receptors but is unable to *Trans*-activate, possibly due to a divergent structure in its extracellular domain [23–26]. However, whether *Cis*-inhibition by other Notch ligands takes place endogenously and how it integrates with *Trans*-activation during development still need to be addressed.

Here, we show that Notch signaling is maintained in prospective neurons, i.e., cells that have completed mitosis but are not yet expressing neuronal differentiation markers. This sustained activity is crucial to allow them to constrict their apical endfoot before they reduce apical junction markers, thus preserving the integrity of the tissue. Moreover, we provide evidence that differentiation is achieved through *Cis*-inhibition of Notch by its ligand Delta-like 1 (Dll1). Finally, we show that the ubiquitin ligase Mindbomb1 (Mib1), by transiently favoring *Trans*-activation at the expense of *Cis*-inhibition in prospective neurons, defers differentiation and allows the tissue to reconcile neuronal commitment with epithelial maintenance.

## Results

### Notch signaling is maintained in prospective neurons

**Fig 1:**
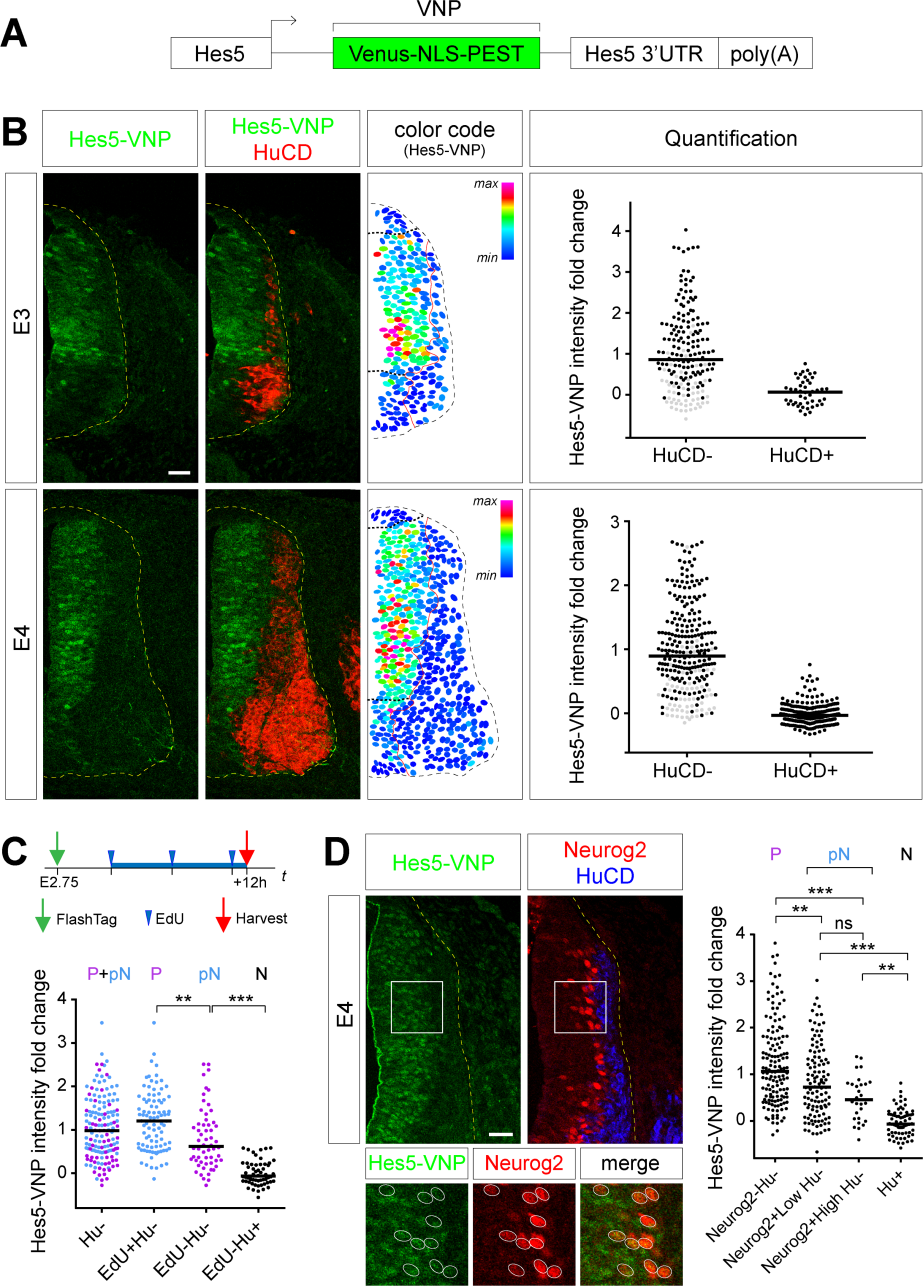
Notch signaling is maintained in prospective neurons. **(A)** Schematic representation of the Hes5-VNP sequence that was inserted in the Notch reporter transgenic chick line. **(B)** Left: Transverse sections of the NT of the Hes5-VNP transgenic line at E3 and E4 immunostained for Venus (green) and HuCD (red) to label neurons. Middle: Color coded map of Hes5-VNP intensity. The red line separates HuCD^−^ from HuCD^+^ cells. The black dotted lines delineate the ventral limit of the roof plate and dorsal limit of the motor neuron domain. Right: Distribution of the Hes5-VNP signal intensity in HuCD^−^ and HuCD^+^ cells. Note that cells within the limits of the black dotted lines of the color code panel were labeled in black in the HuCD^−^ population. **(C)** Top: Time course of the protocol. Bottom: Distribution of the Hes5-VNP signal intensity in FT^+^/HuCD^−^ cells. This population is then divided into EdU^+^ (blue) and EdU^−^ (magenta) cells. A minimum of 58 cells collected from four embryos were analyzed for each group. **(D)** Left: Transverse sections of the dorsal NT in the Hes5-VNP transgenic line at E4 immunostained for Venus (green), Neurog2 (red), and HuCD (blue). Bottom: Enlarged view of the boxed area showing representative examples of Neurog2+ cells. Right: Distribution of the Hes5-VNP signal intensity in Neurog2^−^ and Neurog2^+^ cells. The latter population was divided based on Neurog2^+^ signal intensity. A minimum of 75 cells collected from six embryos were analyzed for each group. Horizontal bars correspond to medians. ns, *p* > 0.05; **p < 0.01, ***p < 0.001 (Kruskal-Wallis test). Underlying data are provided in S1 Data. Scale bar represents 25 μm. See also S1 and S2 Figs. E, embryonic day; EdU, 5-ethynyl-2′-deoxyuridine; FT, FlashTag; Hes5, Hairy and Enhancer of Split 5; HuCD, neuron-specific RNA-binding proteins HuC and HuD; Neurog2, Neurogenin 2; ns, nonsignificant; NT, neural tube; VNP, Venus-NLS-PEST.

Following the completion of mitosis, prospective neurons remain attached to the ventricular surface for a transition period of up to 20 h before they eventually retract their apical endfoot as they start expressing the early differentiation marker class III β-tubulin (Tuj1) [1, 27]. While it is accepted that Notch activity is switched off in differentiated cells, the state of signaling during the transition period that precedes has never been explored. We decided to address this point in a chicken transgenic line carrying a fluorescent reporter of Notch activity. A transgene containing the promoter of the Hairy and Enhancer of Split 5 (Hes5) gene (a target of the Notch pathway) upstream of a destabilized nuclear Venus coding sequence (Venus-NLS-PEST [VNP]) [28] was inserted into the chicken genome (Fig 1A, and see Materials and methods). We first investigated the intensity of the VNP signal through immunostaining (the native VNP signal does not allow direct visualization) in normal conditions. Hes5-VNP distribution was consistent with the endogenous chicken Hairy and Enhancer of Split 5.1 (cHes5.1) expression at embryonic day (E) 4 (S1A Fig, [29]), while nuclear localization of the VNP signal provided a better cellular resolution. Transverse sections of the spinal cord were analyzed during early neurogenesis (E3 and E4), and VNP signal intensity was compared between progenitors and neurons (Fig 1B, the red line delimits the boundary of the differentiated zone in the color code panel). While progenitors displayed a wide spectrum of VNP intensities, all neurons (identified by the expression of the neuron-specific RNA-binding proteins HuC and HuD [HuCD]) showed low VNP levels. This is consistent with data obtained in the mouse cortex using a Hes1 reporter suggesting that Notch target gene expression oscillates in progenitors and is switched off during differentiation [3].

We next assessed whether VNP intensities would reliably reflect perturbations of Notch signaling activity. Notch gain-of-function through overexpression of the Notch intracellular domain (NICD) resulted in a 6-fold increase in VNP intensities as well as a blockade of differentiation (S1B Fig). Conversely, incubation of NT explants with the Notch signaling inhibitor N-(3,5-difluorophenylacetyl-L-alanyl)-S-phenylglycine t-ButylEster (DAPT) led to a rapid reduction of the VNP signal, suggesting a half-life of the reporter of less than 4 h, reaching down to the background level measured in neurons within 6 h of incubation (S1C Fig). Thus, the Hes5-VNP chicken line appears as an excellent tool to monitor the dynamics of Notch signaling in the embryonic spinal cord. Progenitors located in the roof plate region, and ventrally up to the dorsal limit of the motor neuron progenitor domain (delimited by the black dotted lines in Fig 1B, Middle) displayed a lower Notch activity compared to the rest of the ventricular zone (VZ) (Fig 1B, Right; cells in those regions are represented by gray dots). This pattern is consistent with previous reports that floor and roof plates are signaling centers displaying low Notch activity, while the reduced Notch levels measured in the motor neuron progenitor domain may be associated with the early and massive motor neuron differentiation process [30].

Then, we sought to characterize the level of Notch activity in prospective neurons. To this end, we first took advantage of the FlashTag (FT) technique, based on the ability of the cell-permeant dye carboxyfluorescein succinimidyl ester (CFSE) to fluorescently label intracellular proteins. Previous experiments in the mouse developing cortex have shown that upon injection in the ventricles, FT dyes preferentially enter progenitor cells undergoing mitosis, offering a convenient means to synchronously label a cohort of dividing cells and track their progeny over different time periods [31]. To validate the technique and calibrate its dynamics in the chick spinal cord, FT was injected in the NT at E2.75 and fluorescence was monitored at different time points. Fifteen minutes after injection, FT^+^ cells’ nuclei were exclusively located near the ventricular surface, and many were positive for phospho-Histone H3, consistent with the preferential labeling of cells undergoing mitosis (S2A Fig). Increasing incubation times (1 h, 4 h) correlated with FT^+^ nuclei being located at progressively more basal positions and no longer in mitotic cells. This indicates that incorporation into mitotic cells was restricted to a short time period after FT injection, allowing the labeling of a cohort of cells that collectively undergo mitosis in a very narrow time window. We then asked whether the progeny of mitotic cells labeled with FT entered S phase or exited the cell cycle and differentiated. Embryos were injected with the FT dye at E2 or E2.75 (respectively before and after the onset of neurogenic divisions). EdU (5-ethynyl-2′-deoxyuridine) was injected 3 h after FT injection and then every 4 h in order to cumulatively label the whole population of cycling cells (S2B Fig). Embryos were harvested at different time points after FT injection and labeled for EdU incorporation and HuCD expression (S2C and S2D Fig). In both conditions, the number of FT^+^/EdU^+^ cells reached a plateau by 12 h after FT injection, indicating a saturating labeling of cycling progenitors with EdU (S2C and S2D Fig). Consistent with the fact that virtually all progenitors undergo symmetric proliferative divisions at E2 (excluding the motor neuron domain, which differentiates earlier than the rest of the NT and was excluded from the analysis), the plateau of FT^+^/ EdU^+^ was close to 100% in embryos injected at E2 (S2D Fig), and no FT^+^/HuCD^+^ cells were found. By contrast, in embryos injected at E2.75, the plateau of FT^+^/EdU^+^ cells remained below 65% (S2D Fig). Thus, about one third of FT^+^ cells remained EdU^−^. Within this population, the proportion of HuCD^+^ neurons increased between 12 h and 16 h (S2C and S2D Fig). Therefore, three populations could be discriminated based on EdU incorporation and HuCD expression: cycling progenitors (EdU^+^/HuCD^−^), prospective neurons (EdU^−^/HuCD^−^), and neurons (EdU^−^/HuCD^+^). We then investigated the level of Notch signaling in these three populations using FT injection in the Hes5-VNP chicken line. Strikingly, levels of Notch activity in EdU^−^/HuCD^−^ prospective neurons remained elevated 12 h after mitosis (with a median of 0.62 and a mean of 0.80 ± 0.09, the average VNP intensities measured in HuCD^−^ and HuCD^+^ cells being normalized to 1 and 0, respectively [Fig 1C]). This sustained activity is not due to inertia of the Venus reporter fluorescence, because DAPT treatment of NT explants results in complete loss of Venus fluorescence within 6 h (S1C Fig). Hence, prospective neurons maintain high Notch signaling activity up to 12 h after they exit the cell cycle and until they enter differentiation.

To strengthen these results, we sought to identify the population of prospective neurons by another means. As proneural genes promote cell cycle exit and neural commitment [8], they are likely to be expressed at high levels in prospective neurons. We focused on the proneural gene Neurogenin 2 (Neurog2), which is widely expressed in the chick spinal cord [32]. Neurog2 was strongly expressed at the basal limit of the VZ but also in scattered cells within the VZ, albeit at lower levels (Fig 1D). Cumulative EdU incorporation and HuCD staining indicated that these two populations, referred to as Neurog2^Low^ and Neurog2^High^, had mostly exited the cell cycle (S2E Fig), while only a fraction had differentiated (S2F Fig). Thus, the vast majority of Neurog2^+^/HuCD^−^ cells correspond to prospective neurons, amongst which, Neurog2^High^ cells are likely to be closer to differentiation (as twice more Neurog2^High^ than Neurog2^Low^ have started to express the differentiation marker HuCD [S2F Fig]). We compared the level of Notch activity in Neurog2^−^/HuCD^−^ cells (which closely match the progenitor population), Neurog2^Low^/HuCD^−^ and Neurog2^High^/HuCD^−^ cells (prospective neurons), and HuCD^+^ neurons. Remarkably, Neurog2 negative, Low, and High populations of HuCD^−^ cells exhibited progressively lower Notch activity but remained above the level measured in the HuCD^+^ neuronal population (Fig 1D).

Taken together, these results indicate that Notch signaling is maintained in prospective neurons until they eventually differentiate. This raises the question of the importance of maintaining Notch activity during the events preceding differentiation.

### Maintenance of Notch signaling is required for proper neuronal delamination

**Fig 2:**
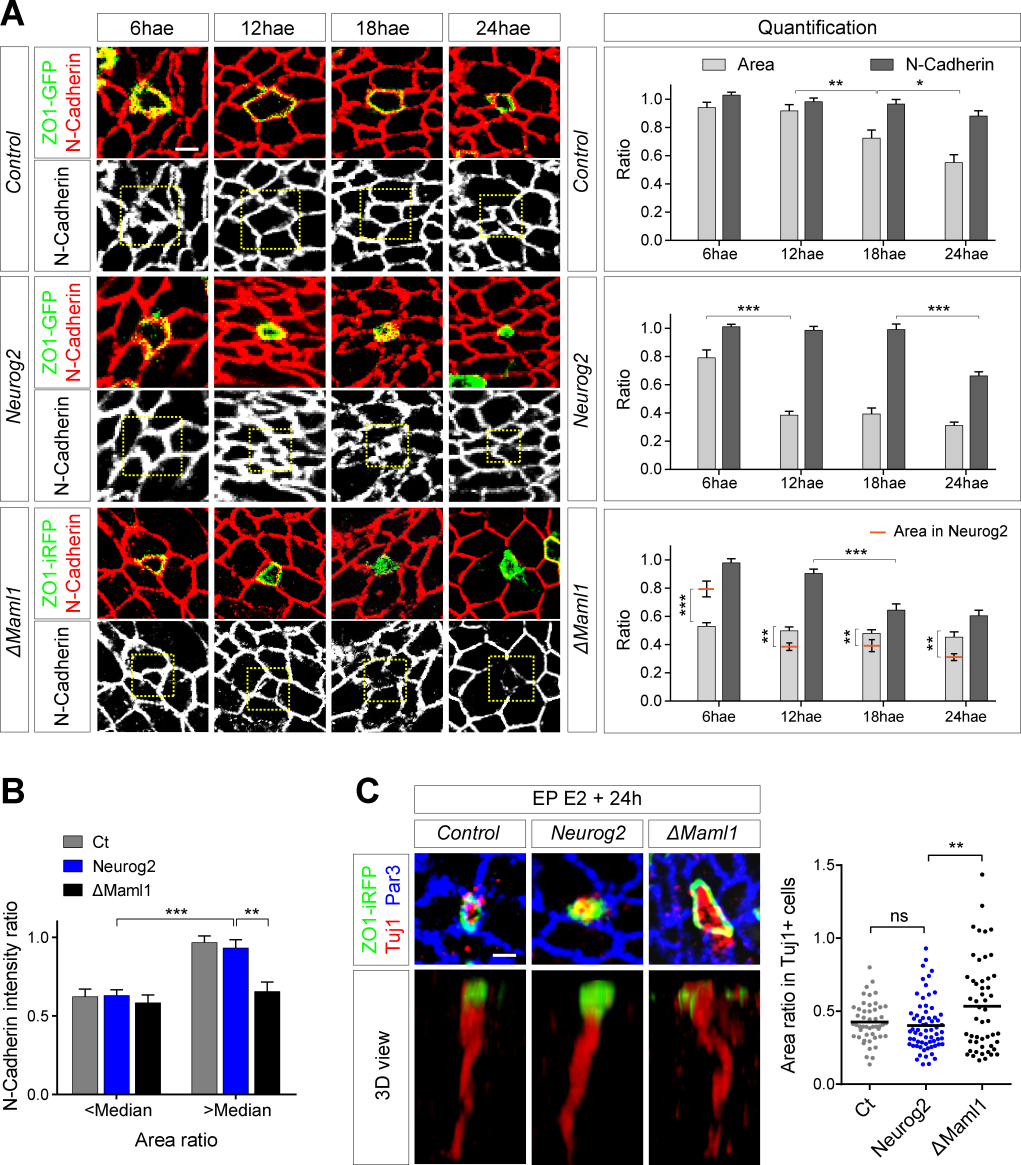
Sequence of events leading to neuron delamination. **(A)** Left: Apical view of the NT electroporated at E2 with ZO1-GFP/iRFP (green), along with the constructs indicated on the left, and harvested at different hae, followed by an immunostaining for N-Cadherin. The boxed area indicates the cell of interest. Right: Quantification of the apical area ratio (ratio of the area of one transfected cell versus the mean area of four of its close non-transfected neighbors) and N-Cadherin level ratio (ratio of the average pixel intensity within the apical circumference of one transfected cell corrected by the background versus the mean of average pixel intensity of four of its close non-transfected neighbors) at different hae. Data represent mean + SEM. **(B)** N-Cadherin intensity ratio as a function of apical area ratio at 24 hae. Data were taken from **(A)**. The “median” used as a threshold to discriminate between small and large apical areas corresponds to the median of the control (0.62). **p < 0.01; ***p < 0.001 (one-way ANOVA). **(C)** Top: Apical view of the NT transfected with ZO1-iRFP (green) along with the indicated constructs and immunostained for Tuj1 (red) and Par3 (blue). Bottom: Three-dimensional view of the cell represented above but showing only the ZO1-iRFP and Tuj1 stainings. Right: Scatterplot of the mean apical area ratio for Tuj1^+^ cells. Each point represents one apical area ratio calculated as in **(A)**. *n* = 49, 66, 51 cells collected from five embryos were analyzed for control, Neurog2, and ΔMaml1, respectively. ns, *p* > 0.05; ***p < 0.001 (Kruskal-Wallis test). Horizontal bars correspond to means. Underlying data are provided in S1 Data. Scale bar represents 2 μm. See also S3 Fig. ΔMaml1, dominant-negative Mastermind-like 1; E, embryonic day; EP, electroporation; GFP, green fluorescent protein; hae, hour after electroporation; iRFP, infrared fluorescent protein; Neurog2, Neurogenin 2; ns, nonsignificant; NT, neural tube; Par3, Partition defective protein 3; ZO1, Zonula Occludens 1.

A hallmark of neuronal differentiation is the withdrawal of the apical attachment from the ventricular surface [1, 2, 27, 33]. To gain insight into the cellular events that accompany this delamination process, we investigated three parameters in parallel: the size of the apical area, the level of N-Cadherin at apical junctions, and the expression of the early differentiation marker Tuj1 at the apical surface (i.e., in nascent neurons that are still attached). These parameters were analyzed at 6, 12, 18, and 24 hours after electroporation (hae), focusing on single electroporated cells surrounded by non-transfected neighbors (the latter were used as a reference for measurements; see Materials and methods). Electroporation targets a mix of cycling progenitors and apically attached prospective neurons. While early time points (6 h, 12 h) will still retain many progenitors, these will eventually divide and appear as pairs that will be discarded from the analysis, such that at later time points (18 h, 24 h), the selected population will be enriched in prospective neurons. In cells transfected with a ZO1-GFP control vector alone, a decrease in the apical area was apparent at 18 hae and was further enhanced at 24 hae (Fig 2A, top panel). We also observed a modest decrease of N-Cadherin levels at 24 hae, which was not significant when considering the whole population (Fig 2A, top panel). However, when “small” (below the median) and “large” (above the median) areas were discriminated at 24 hae, we observed a significantly lower level of N-Cadherin in cells with a small apical area (Fig 2B). Moreover, the differentiation marker Tuj1 was almost exclusively expressed in this population (Fig 2C). These results are consistent with a differentiation process, as apical constriction and N-Cadherin reduction are features associated with neuronal delamination [1, 2, 27, 33].

To characterize the evolution of these parameters over time more specifically in cells committing to differentiation, we sought to synchronize the differentiation process. To this end, cells were transfected with the proneural gene Neurog2. As previously reported [34–36], Neurog2 repressed the expression of the progenitor marker Paired box gene 6 (Pax6) (S3A Fig), induced cell cycle exit after 24 h (S3B Fig), and strongly increased the differentiation rate at 48 hae (S3D Fig). In this case, we observed a strong reduction of the apical area from 12 hae, while reduction of N-Cadherin was detected only at 24 hae (Fig 2A, middle panel). In addition, reduction of N-Cadherin levels and expression of Tuj1 were observed almost exclusively in cells with a “small” area 24 hae (Fig 2B and 2C). Thus, control and Neurog2-induced differentiating cells appear to follow a similar sequence of events: constriction of the apical area precedes the reduction of N-Cadherin levels at apical junctions and the expression of Tuj1. Importantly, 24 h after Neurog2 electroporation, when most electroporated cells have exited the cell cycle (S3B Fig), the majority of Neurog2^+^/HuCD^−^ cells correspond to prospective neurons, and accordingly, these cells retained high levels of Notch reporter expression (S3C Fig).

We next wanted to assess the role of Notch signaling in this context. To this end, we measured these same parameters in cells transfected with a dominant negative version of the Notch pathway transcriptional coactivator Mastermind-like 1 (ΔMaml1) [37, 38]. In contrast to Neurog2, ΔMaml1 directly inhibits Notch transcriptional targets. Consistent with this, transfection of ΔMaml1 induced a massive decrease of Notch activity at 24 hae in the HuCD^−^ population and pushed cells to differentiate faster than Neurog2 (S3E and S3F Fig). These cells reduced their apical area ratio and their N-Cadherin level earlier than in the Neurog2 situation (Fig 2A, bottom panels). However, while the constriction of the apical surface appeared earlier (6 hae), at later time points the average apical surface remained significantly larger than in the Neurog2 case (Fig 2A, 12, 18, and 24 hae, apical surface values for Neurog2 were inserted in the ΔMaml1 graph for comparison). Moreover, unlike in the control and Neurog2 situations, low N-Cadherin levels were no longer restricted to cells with a small apical surface (Fig 2B) and Tuj1-positive nascent neurons with abnormally large apical domains were observed (Fig 2C). Taken together, these data suggest that upon precocious blockade of Notch signaling, N-Cadherin reduction and neuronal differentiation occur before apical constriction is complete.

**Fig 3:**
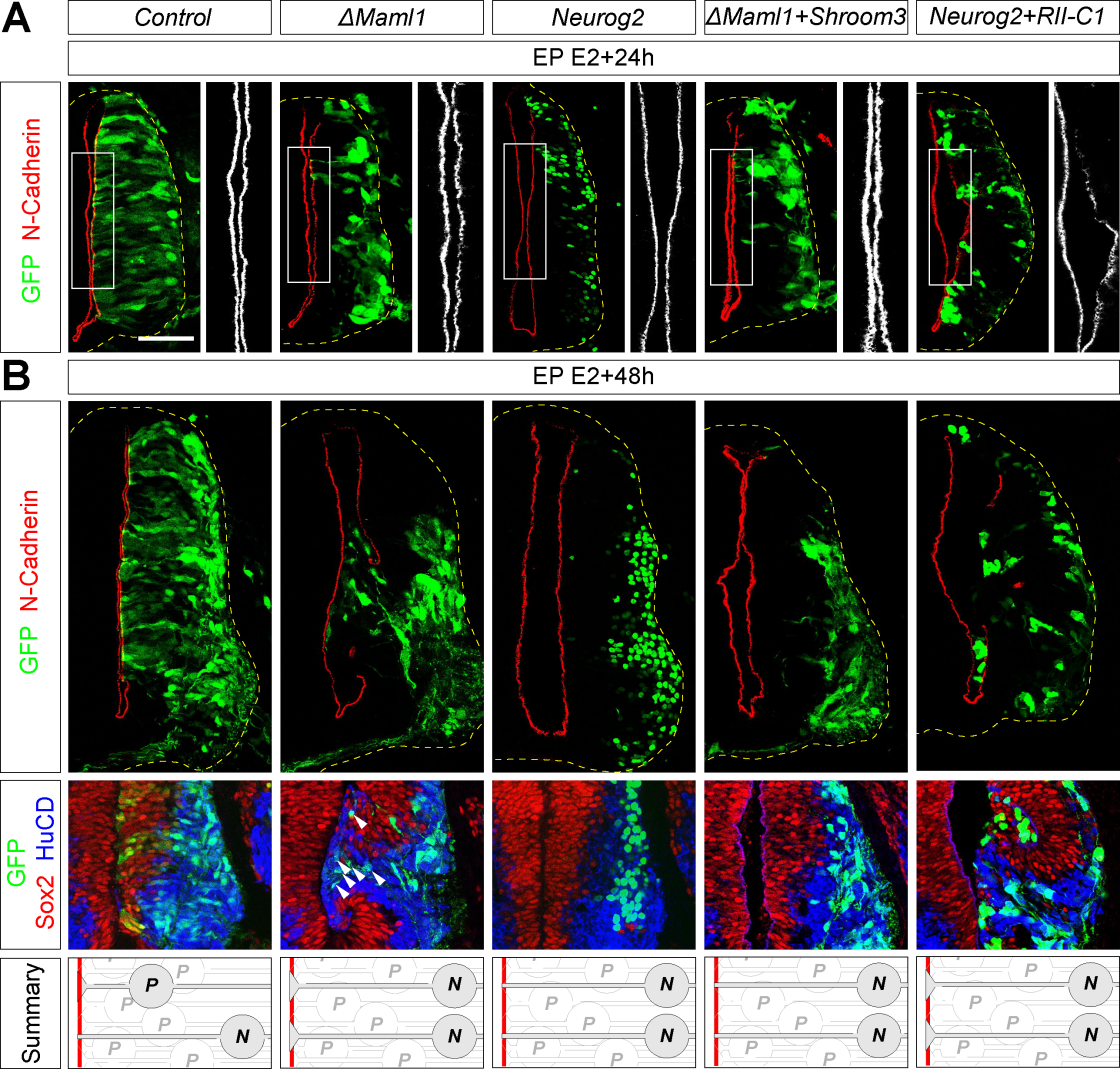
Effects of Notch signaling and apical constriction modulators on apical markers and tissue integrity. **(A)** Transverse sections of the NT transfected at E2 with the indicated constructs, harvested at E3 and immunostained for N-Cadherin (red). **(B)** Transverse sections of the NT transfected at E2 with the indicated constructs, harvested at E4 and immunostained for N-Cadherin (red); and for Sox2 (red) and HuCD (blue) to label progenitors and neurons, respectively. Transfection is reported by GFP expression. Summary: Schematic of the effects observed on tissue integrity. Gray cells correspond to electroporated cells. Scale bar represents 50 μm. See also S4 Fig. ΔMaml1, dominant-negative Mastermind-like 1; E, embryonic day; EP, electroporation; GFP, green fluorescent protein; HuCD, neuron-specific RNA-binding proteins HuC and HuD; N, neuron; *Neurog2*, *Neurogenin 2*; NT, neural tube; P, progenitor; *RII-C1*, *Shroom3 binding site on ROCK2*; *Shroom3*, *Shroom family member 3*; Sox2, SRY (sex determining region Y) box 2.

We then investigated whether the effects observed at the single cell level would have a global impact on the integrity of the NT. Very strikingly, in contrast to control and Neurog2 situations, ΔMaml1 overexpression led to a noticeable decrease of all apical markers analyzed on transverse views at 24 hae (Fig 3A and S4B Fig). This resulted one day later in a severe disruption of the ventricular wall associated with the presence of ectopic neuronal masses protruding into the spinal cord lumen (Fig 3B). Remarkably, only a fraction of these ectopic neurons corresponded to transfected cells (Fig 3B, see arrowheads), suggesting that the down-regulation of apical markers in the transfected population was sufficient to induce a massive disorganization at the tissue scale. While blocking Notch activity results in a decrease of apical markers all along the dorsal–ventral axis at 24 hae (Fig 3A and S4B Fig), breaches in the ventricular wall were observed one day later almost exclusively in the ventral region of the NT. Motor neurons are the first neurons to be detected in the spinal cord (at E2) and are already extensively differentiated at E3 in the ventral NT. This suggests that a large population of nascent motor neurons has collectively delaminated between E2 and E3, which may render the ventral NT more sensitive to a weakening of the apical network.

The down-regulation of apical markers following Notch blockade is correlated with the presence of differentiating cells displaying large apical domains (Fig 2C). This suggests that in the control situation (or in Neurog2 expressing cells), apical constriction may help to confine low N-Cadherin levels to only small fractions of the apical junction network and contribute to preserving epithelial integrity when neurons delaminate. To functionally test this hypothesis, we sought to alter the size of the apical area in differentiating cells. Apical constriction was shown to rely on actomyosin contraction and is regulated by Rho-GTPases family members [39]. A typical example of apical constriction is observed in the neurulation process, during which the actin-binding protein Shroom family member 3 (Shroom3) induces apical constriction by recruiting Rho kinases (ROCKs) to adherens junctions [40]. We found that overexpression of Shroom3 forced apical constriction. Conversely, a fragment of ROCK2 designated as RII-C1 (Shroom3 binding site on ROCK2) shown to dominantly interfere with the interaction between endogenous full-length ROCK2 and Shroom3 led to an increase in apical areas, suggesting that Shroom family members are active at neurogenic stages and regulate the size of the apical footprint of neuroepithelial cells (S4C and S4D Fig). Co-transfection of Shroom3 with ΔMaml1 strongly reduced the apical area, bringing it down to the value measured in the Neurog2 situation, and increased N-Cadherin apical level (S4C and S4E Fig). Strikingly, this rescued the ΔMaml1 phenotypes: apical markers distribution was restored at 24 hae (Fig 3A, S4B Fig) and tissue integrity was no longer affected at 48 hae (Fig 3B). By contrast, inhibiting Shroom-ROCK2 interaction in Neurog2 transfected cells through overexpression of the RII-C1 fragment led to an increase of the apical area (S4C and S4D Fig), which correlated with a decrease of apical markers on transverse (S4B Fig) and apical views (S4C and S4E Fig), mimicking the ΔMaml1 overexpression phenotype. Consistently, this was followed by a disruption of the ventricular wall at 48 hae (Fig 3B), while Shroom3 and RII-C1 alone had no effect on apical marker localization (S4B Fig).

Taken together, these results suggest that sustained Notch activity is necessary in prospective neurons to allow reduction of the apical size to take place before apical junction markers are down-regulated and neurons delaminate, therefore preserving the integrity of the ventricular wall.

### Dll1 levels control differentiation through the regulation of Notch activity

Having shown that maintenance of Notch signaling is critical during the last steps leading to differentiation, we next investigated the mechanisms regulating the level of Notch activity during this transition. Increase in proneural gene expression is known to be required for differentiation and is correlated with a reduction of Notch activity. However, the connection between these two events remains to be clarified. In the chick spinal cord, the Notch ligand Dll1 is an early target of Neurog2 [35], and functional approaches in the mouse cortex suggested that Dll1 expression was necessary and sufficient for neural differentiation [14]. We first investigated the role of Dll1 on neurogenesis in the chick spinal cord. Consistent with published results, we observed a strong increase in the differentiation rate of Dll1 transfected cells 48 hae (S5A Fig). By contrast, down-regulation of Dll1 following mosaic electroporation of a short hairpin RNA (shRNA) against chick Dll1 [41] reduced differentiation (S5B Fig). We then used the Hes5-VNP transgenic line to investigate the level of Notch activity following gain and loss of Dll1 function, focusing on HuCD^−^ undifferentiated cells. Consistent with their impact on differentiation, gain and loss of Dll1 function led to a decrease and an increase of Notch activity, respectively (S5C and S5D Fig). It should be noted that Dll1 is widely expressed in the spinal cord except for the dorsal dI6 and intermediate V1 interneuron domains. As Notch activity and differentiation rate following Dll1 misexpression were analyzed in the dorsal and intermediate regions of the NT irrespective of the endogenous expression of Dll1, our results may be slightly underestimated.

### Mib1 blocks the ability of Dll1 to *Cis*-inhibit Notch signaling

**Fig 4:**
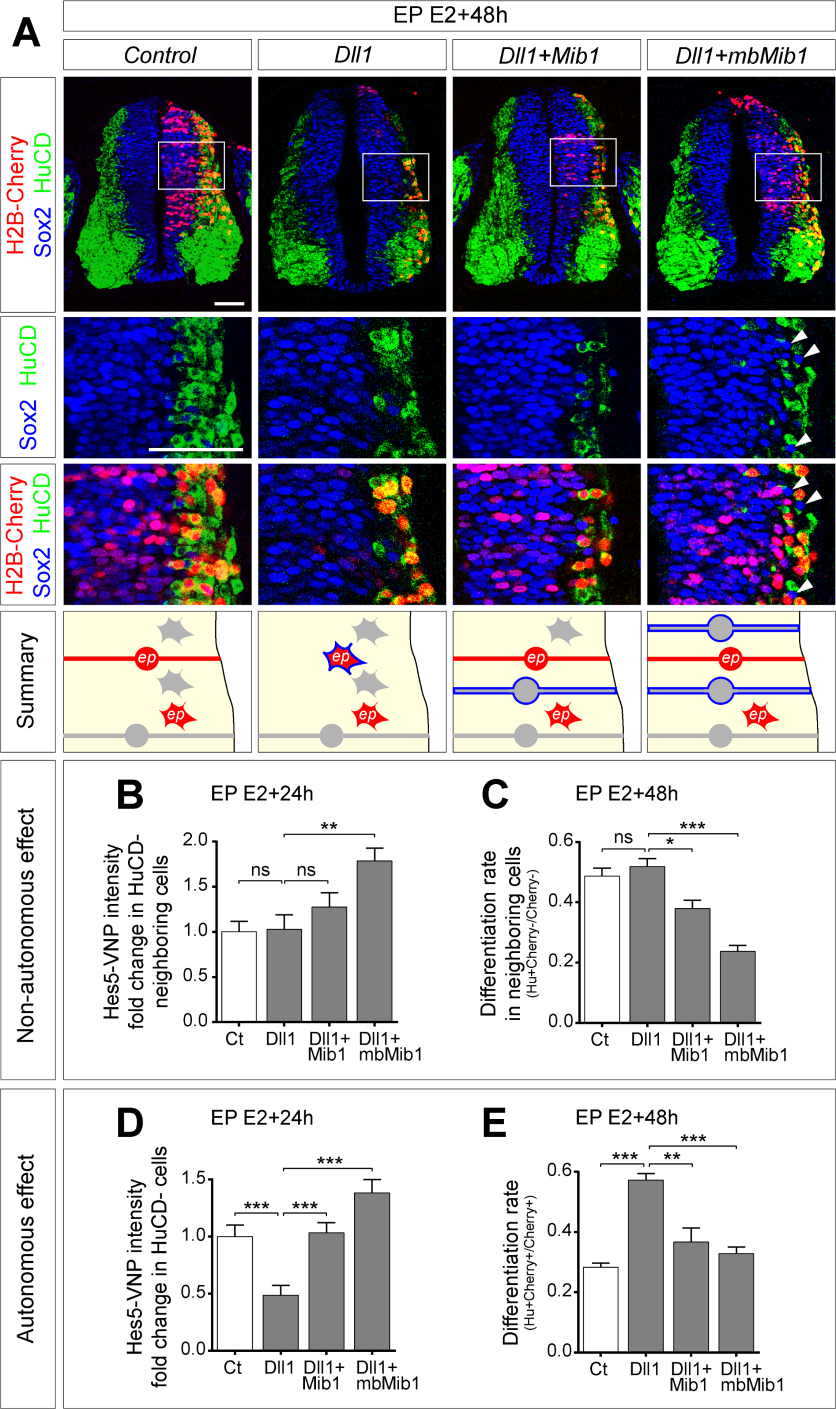
Mib1 blocks the ability of Dll1 to *Cis*-inhibit Notch signaling. **(A)** Top: Transverse sections of the NT transfected at E2, with the indicated constructs and harvested at E4. Immunostaining for Sox2 (blue) and HuCD (green) labels progenitors and neurons, respectively. Transfection is reported by H2B-Cherry expression. Arrowheads indicate ectopic Sox2^+^ progenitors adjacent to HuCD^+^ transfected neurons. Bottom: Summaries of the effects of Dll1 and Mib1 on neurogenesis. Red and gray cells correspond to electroporated (ep) and non-electroporated cells, respectively. Round and star-shaped cells correspond to progenitors and neurons, respectively. Blue outlines indicate cells changing fate, autonomously or non-autonomously, in each condition. **(B, D)** Quantification of the Hes5-VNP signal intensity in HuCD^−^ cells either **(B)** non-transfected (surrounded by at least four transfected cells) or **(D)** transfected 24 hae with the indicated constructs. Data represent fold change compared to control. **(B)** *n* = 54, 35, 35, 54 cells were analyzed for control, Dll1, Dll1+Mib1, and Dll1+mbMib1, respectively. **(D)** *n* = 58, 59, 59, 66 cells were analyzed for control, Dll1, Dll1+Mib1, and Dll1+mbMib1, respectively. Data were collected from four to six embryos for each experimental group. ns, *p* > 0.05; **p < 0.01; ***p < 0.001 (Kruskal-Wallis test). **(C, E)** Quantification of the differentiation rate in **(C)** non-transfected neighbors (number of non-transfected HuCD^+^ cells adjacent to a HuCD^+^ transfected cell on the total number of adjacent cells) or **(E)** transfected cells (number of HuCD^+^ cells on total transfected cells) 48 hae with the indicated constructs. Data represent mean + SEM. *n* = 14 (6 embryos), 10 (8 embryos), 14 (6 embryos), 18 (6 embryos) sections were analyzed for control, Dll1, Dll1+Mib1, and Dll1+mbMib1, respectively. ns, *p* > 0.05; *p < 0.05; **p < 0.01; ***p < 0.001 (one-way ANOVA). Analyses were performed on the same sections for **(B)** and **(D)**, and for **(C)** and **(E)**. Underlying data are provided in S1 Data. Scale bar represents 50 μm. See also S5 Fig. Ct, control; Dll1, Delta-like 1; E, embryonic day; ep, electroporated; hae, hour after electroporation; Hes5, Hairy and Enhancer of Split 5; HuCD, neuron-specific RNA-binding proteins HuC and HuD; H2B-Cherry, Histone 2B fused to Cherry; mbMib1, Mib1 constitutively tethered to the plasma membrane; Mib1, Mindbomb1; ns, nonsignificant; NT, neural tube; Sox2, SRY (sex determining region Y) box 2; VNP, Venus-NLS-PEST.

Dll1 expression in a differentiating cell could lead to reduced Notch activity either indirectly by *Trans*-activation of Notch signaling in the neighbors that would therefore not *Trans*-activate in return (according to the lateral inhibition with feedback model) or directly through *Cis*-inhibition of Notch receptors in the same cell [20]. However, answering this question in vivo cannot be obtained solely by Dll1 misexpression, which would impact both *Trans* and *Cis* phenomena. Thus, we decided to take advantage of the ability of the ubiquitin ligase Mindbomb1 (Mib1) to promote Notch *Trans*-activation. We first tested the ability of Dll1 alone or with Mib1 to induce *Trans*-activation of Notch signaling in undifferentiated cells (HuCD-negative) 24 h after transfection. To this end, the intensity of the Hes5-VNP reporter was measured in non-transfected “neighbor cells” contacted by a minimum of four transfected cells. Dll1 alone was unable to *Trans*-activate signaling in neighbors (Fig 4B). In contrast, our measures upon Mib1 co-transfection indicated a trend towards increased Notch activity, although it failed to reach statistical significance (Fig 4B). We recently reported that in normal conditions, Mib1 is strongly enriched at the centrosome and barely detectable at the membrane in the NT, suggesting that only a fraction of it interacts with Dll1 [27]. To potentiate this interaction, we engineered a version of Mib1 constitutively tethered to the plasma membrane (mbMib1) by addition of an N-terminal myristoylation sequence. Remarkably, co-transfection of Dll1 with mbMib1 resulted in a significant increase of Notch activity in neighbor cells (Fig 4B). We reasoned that this higher Notch activity in neighbors should hinder their ability to differentiate. Indeed, while Dll1 alone had no impact on the differentiation rate of neighboring cells, the latter was consistently reduced following co-transfection of Mib1 and mbMib1 (Fig 4A and 4C). These data suggest that endogenous Mib1 is limiting and that Dll1 can *Trans*-activate the Notch pathway only when co-transfected with Mib1.

We then analyzed the same parameters in transfected cells. Dll1 alone led to a noticeable decrease of Notch activity 24 hae in HuCD^−^ cells (Fig 4D), accompanied by an increased differentiation rate 48 hae (Fig 4A and 4E). If this effect was relying on a feedback-based lateral inhibition mechanism, as it was previously proposed, one would expect Mib1 to enhance the phenotype observed with Dll1 by promoting *Trans*-activation in neighbors. On the contrary, we observed that Mib1 and mbMib1 induced an increase of Notch signaling (Fig 4D) and a reduction of the differentiation rate compared to Dll1 alone (Fig 4A and 4E).

Taken together, these results indicate that Dll1 overexpression promotes differentiation of neural progenitors cell autonomously through *Cis*-inhibition of Notch signaling and that Mib1 is able to block this effect by converting Dll1 from a *Cis*-inhibiting to a *Trans*-activating ligand.

### Mib1 blocks Notch *Cis*-inhibition to defer differentiation and preserve neuroepithelial integrity

**Fig 5:**
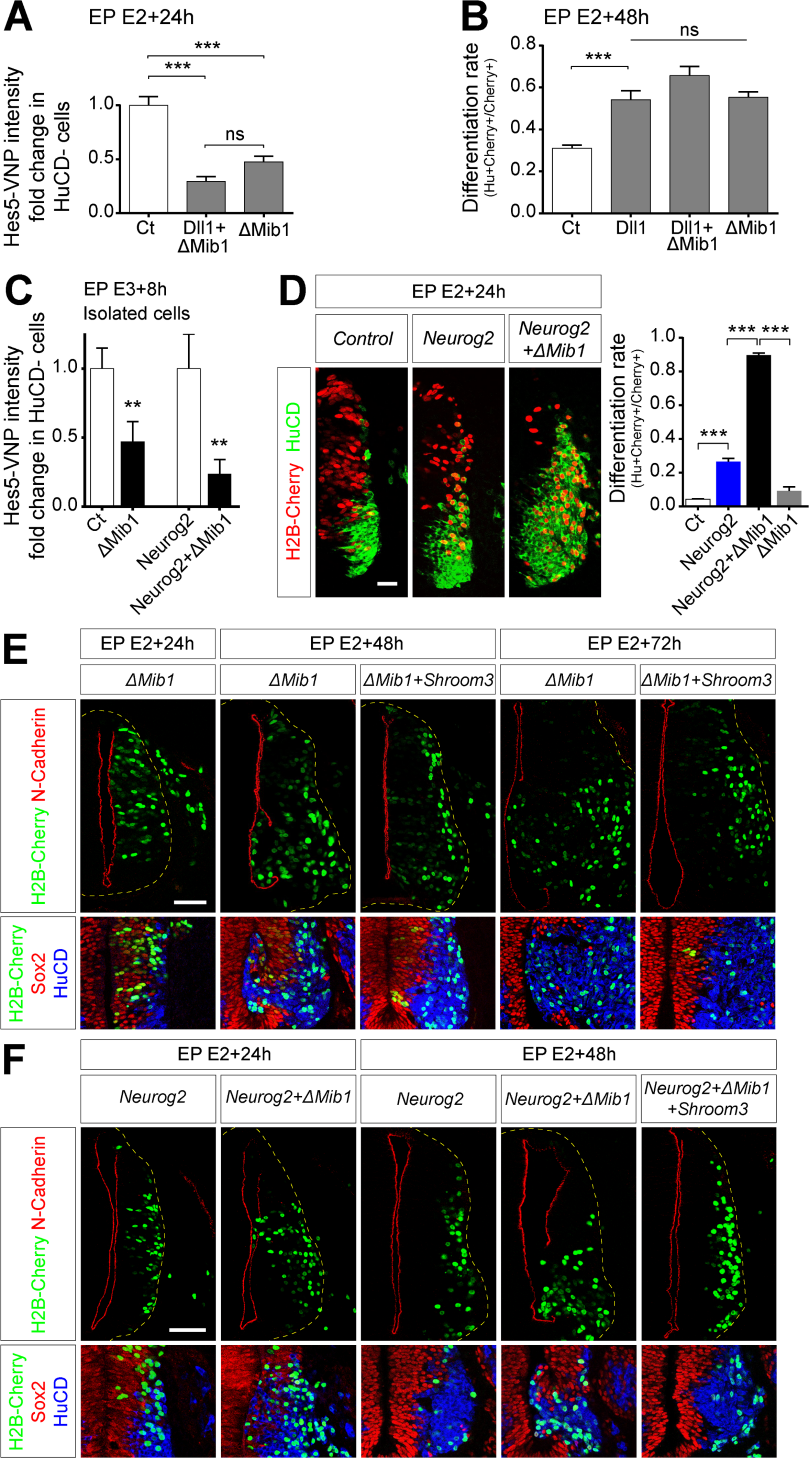
Mib1 blocks Notch *Cis*-inhibition to defer differentiation and preserve neuroepithelial integrity. **(A)** Quantification of Hes5-VNP intensity in HuCD^−^ cells transfected with the indicated constructs at E2 and harvested 24 hae. Data represent fold change compared to control. A minimum of 108 cells were analyzed for each group. ns, *p* > 0.05; ***p < 0.001 (Kruskal-Wallis test). **(B)** Quantification of the differentiation rate (number of HuCD^+^ cells on total transfected cells). Data represent mean + SEM. *n* = 12 (3 embryos), 13 (6 embryos), 15 (6 embryos), 12 (4 embryos) sections were analyzed for control, Dll1, Dll1+ΔMib1, and ΔMib1, respectively. ns, *p* > 0.05; ***p < 0.001 (one-way ANOVA). **(C)** Quantification of the Hes5-VNP signal intensity in HuCD^−^ and isolated cells transfected at E3 with the indicated constructs at low voltage (15 V) and harvested 8 h later. Data represent fold change compared to control. *n* = 37 (3 embryos), 31 (7 embryos), 15 (3 embryos), and 25 (4 embryos) cells were analyzed for control, ΔMib1, Neurog2, and Neurog2+ΔMib1, respectively. **p < 0.01 (Mann-Whitney *U* test). **(D)** Left: Transverse sections of the NT transfected at E2 with the indicated constructs, harvested at E3 and immunostained for HuCD (green) to label neurons. Transfection is reported by H2B-Cherry expression. Right: Quantification of the differentiation rate (number of HuCD^+^ cells on total transfected cells). Data represent mean + SEM. For 24 hae, *n* = 14, 12, 19, 7 sections collected from six embryos for each experimental group were analyzed for control, Neurog2, Neurog2+ΔMib1, and ΔMib1, respectively. ***p < 0.001 (one-way ANOVA). Underlying data are provided in S1 Data. **(E, F)** Transverse sections of the NT transfected at E2 with the indicated constructs and immunostained for N-Cadherin (red); and for Sox2 (red) and HuCD (blue) to label progenitors and neurons, respectively. Transfection is reported by H2B-Cherry expression (green). Scale bar represents 50 μm. ΔMib1, dominant-negative Mib1; Ct, control; Dll1, Delta-like 1; E, embryonic day; EP, electroporation; hae, hour after electroporation; Hes5, Hairy and Enchancer of Split 5; HuCD, neuron-specific RNA-binding proteins HuC and HuD; H2B-Cherry, Histone 2B fused to Cherry; Mib1, Mindbomb1; Neurog2, Neurogenin 2; ns, nonsignificant; NT, neural tube; *Shroom3*, shroom family member 3; Sox2, Sox2, SRY (sex determining region Y) box 2; VNP, Venus-NLS-PEST.

We then sought to address whether *Cis*-inhibition of the Notch pathway by endogenous ligands occurs in the neuroepithelium. The above results suggest that Mib1 may promote Notch response not only in signal-receiving neighbors through *Trans*-activation but also in the signal-sending cell by blocking the *Cis*-inhibition process. To test this, we interfered with Mib1 function using a dominant negative version lacking its ring finger domain (ΔMib1) [42], which retains the interaction with Delta ligands but is unable to promote their maturation and endocytosis. Blocking Mib1 activity should therefore enhance *Cis*-inhibition and reduce Notch signaling cell autonomously. Indeed, overexpression of ΔMib1 reduced Notch activity (Fig 5A) and increased differentiation (Fig 5B), thus mimicking the effects of Dll1 alone, while co-electroporation of Dll1 and ΔMib1 did not significantly enhance the effect of either construct. However, blocking Mib1 function in a massive manner is also likely to alter Notch *Trans*-activation among contacting neighbor and sister cells. Thus, to restrict our analysis to isolated cells, embryos were electroporated under clonal conditions at E3 in order to target cells during the neurogenic peak and harvested shortly after (8 h) to minimize the probability of cell division. Clonal inhibition of Mib1 resulted in a significant decrease of Notch activity in electroporated cells as early as 8 hae (Fig 5C), providing strong evidence that *Cis*-inhibition takes place endogenously in the vertebrate nervous system.

While Mib1 blockade reduces Notch activity (Fig 5A and 5C), unlike Neurog2 it is not sufficient to rapidly force cells to exit the cell cycle and differentiate. However, it is essential for the process of asymmetric division and Mib1 loss-of-function will increase neurogenesis on a longer term [27]. Consistent with this, differentiation was only mildly increased at 24 hae compared to Neurog2 overexpression (Fig 5D), with no effect on N-Cadherin levels (Fig 5E). However, longer incubation times resulted in more neurons induced (Fig 5B) and large breaches in the ventricle (Fig 5E), suggesting that Mib1 regulates both the differentiation rate and the delamination process. Importantly, Shroom3 co-expression rescued NT morphology at 48 and 72 hae (Fig 5E). These results suggest that Mib1-dependent Notch maintenance is required to regulate the pace of differentiation and to allow proper neuronal delamination.

To bypass the effects of Mib1 in binary fate decisions and further characterize its function in the delamination and differentiation of prospective neurons, we performed similar experiments in cells also expressing Neurog2. ΔMib1 and Neurog2 co-expression led to a sharp decrease of Notch activity in prospective neurons at 8 hae (Fig 5C) and to a dramatic increase in differentiated HuCD^+^ cells at 24 hae compared to ΔMib1, Neurog2, or even ΔMaml1 alone (compare Fig 5D with S3F Fig). We then assessed the localization of apical markers at different times following transfection of Neurog2 and/or ΔMib1 (Fig 5E and 5F). Whereas neither ΔMib1 nor Neurog2 alone had any effect, N-Cadherin level was reduced upon co-expression at 24 hae, and breaches along the ventricular wall could be observed one day later and occasionally as early as 24 hae (Fig 5F). Moreover, co-transfection of Shroom3 rescued the morphology of the NT at 48 hae (Fig 5F).

Mib1 was previously shown to control the rate of neurogenesis in vertebrates by promoting Notch *Trans*-activation [43]. Our results suggest that Mib1 promotes Notch activity not only through *Trans*-activation in signal-receiving neighbors but also in the signal-sending cell by blocking the *Cis*-inhibition process. Overall, our data indicate that Mib1 actively sustains Notch signaling in prospective neurons to regulate the pace of differentiation and to allow proper neuronal delamination.

## Discussion

**Fig 6:**
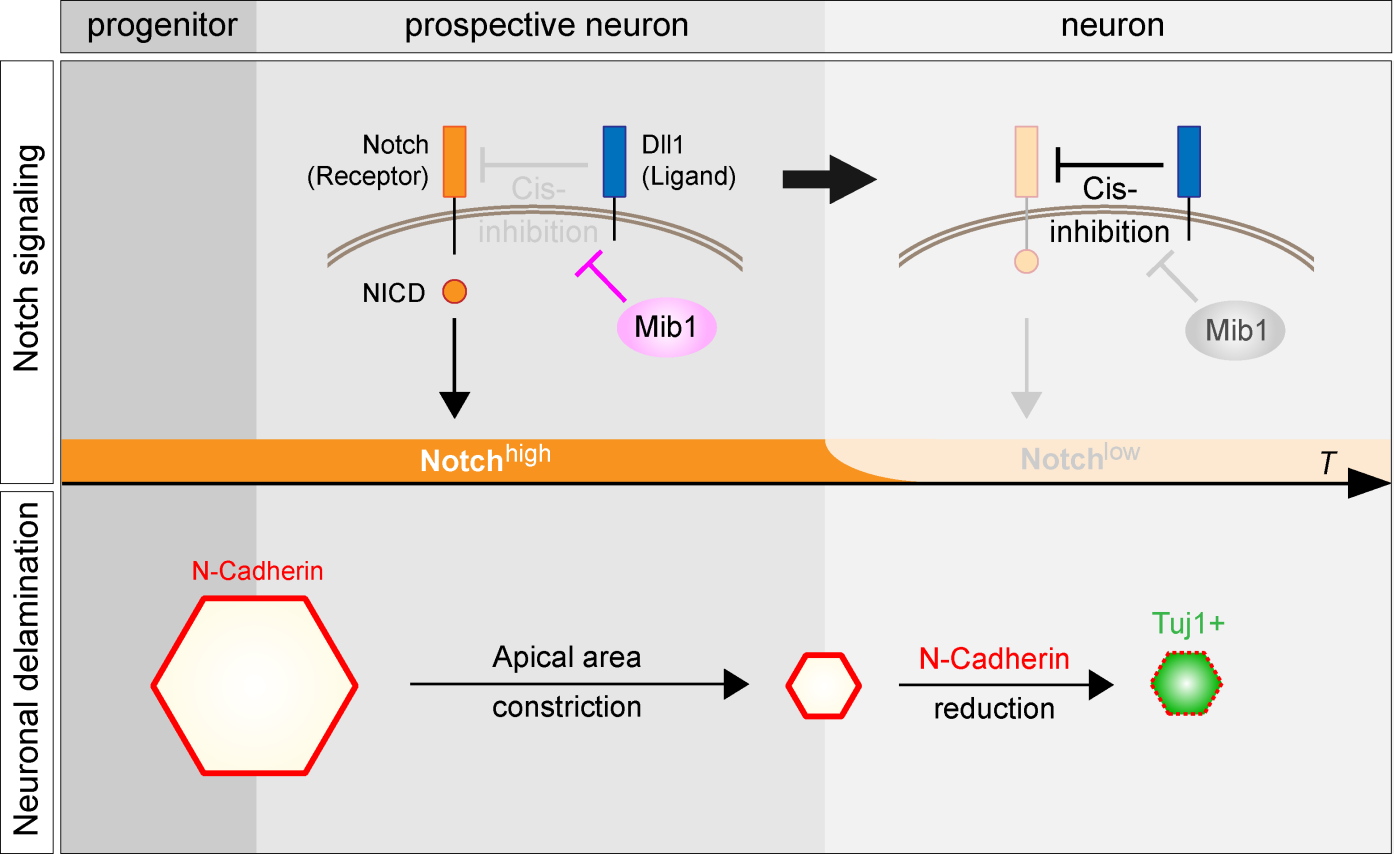
Model for the role of Mib1-dependent Notch activity in the regulation of neuronal delamination. Top: Prospective neurons maintain a high level of Notch activity until they fully differentiate. Mib1 is required during that transition phase to keep Dll1 from *Cis*-inhibiting the Notch receptor. This allows Notch to be *Trans*-activated by Dll1 present on neighboring cells (not represented here), resulting in the release of the NICD. When the Dll1/Mib1 ratio is sufficiently high, *Cis*-inhibition takes place and Notch activity is rapidly turned off. Bottom: Sustained Notch activity allows prospective neurons to shrink their apical area and keeps them from differentiating. As Notch activity is decreased, N-Cadherin levels are down-regulated and neuronal differentiation markers start being expressed. Dll1, Delta-like 1; Mib1, Mindbomb1; NICD, Notch intracellular domain; *T*, time.

Taken together, our results suggest a model in which the regulation of Notch *Cis*-inhibition through the interplay between Dll1 and Mib1 allows prospective neurons to delaminate from the ventricle while preserving the integrity of the NT (Fig 6). Following mitotic exit, prospective neurons maintain a high level of Notch activity until they start expressing neuronal markers. During that transition period, they first contract their apical domain and later reduce their level of N-Cadherin. Hence, apical adhesion is reduced only in restricted areas of the ventricular surface, making final delamination compatible with the preservation of the apical junctional network. Moreover, we show that the maintenance of Notch activity in prospective neurons relies on the ability of Mib1 to block the *Cis*-inhibitory activity of Dll1.

The transition period that separates the mitotic exit of prospective neurons from the appearance of the earliest differentiation markers is at the moment poorly defined. It was suggested that the proneural gene Neurog2 induces an early cell cycle arrest later followed by an irreversible cell cycle exit associated with differentiation [35]. Using a Notch reporter chick line, we provide evidence that Notch signaling remains elevated until prospective neurons have differentiated, suggesting that sustained Notch activity is compatible with cell cycle arrest. Although Notch signaling is classically associated with a proliferative and undifferentiated status, several lines of evidence challenge this view. Remarkably, we found that Notch gain-of-function strictly kept Neurog2 from inducing differentiation but did not prevent Neurog2-induced cell cycle arrest (S6A and S6B Fig). Consistent with this, Neurog2 was documented to drive cell cycle exit and differentiation independently [35]. Moreover, in the adult mouse Notch is required to maintain neural stem cells in a quiescent and undifferentiated state and keep them from proliferating [44]. Taken together, these results suggest that Notch signaling is a powerful guardian of the undifferentiated state but is not necessarily associated with a proliferative behavior.

Furthermore, we show that maintaining Notch activity in prospective neurons is necessary for neuronal delamination to take place properly. The principles that underlie neuronal delamination have only started to be investigated. While N-Cadherin reduction is a mandatory event of the delamination process in the spinal cord [2], we show that this down-regulation needs to be preceded by a reduction of the apical area. Apical constriction is blocked by a dominant negative construct (RII-C1) that was shown to hinder the interaction between Shroom3 and ROCK2 (S4 Fig, [40]). The RII-C1 construct may affect the activity of other Shroom family members that are also expressed in the NT and can drive apical constriction under certain conditions [45, 46]. While Shroom3 is a key regulator of apical constriction previously involved in various morphogenetic events [40, 47, 48], we implicate here for the first time a Shroom-like activity in a delamination process. Interestingly, Shroom blockade results in increased apical areas in all transfected cells (S4C and S4D Fig), suggesting it plays a role in cycling progenitors to control the stability of the apical surface and becomes more active as cells commit to differentiation. This profile makes Shroom family members good candidates to be direct targets of Neurog2. Consistent with this possibility, the levels of Shroom1 and 3 transcripts were up-regulated within 6 h by Neurog2 overexpression in the chick NT (personal communication, S. Bel-Vialar). By contrast, down-regulation of N-Cadherin in the spinal cord involves a transcriptional relay through expression of the Forkhead transcription factors FoxP2 and FoxP4 (FoxP2/4) transcription factors acting downstream of Neurog2 [2]. Thus, Neurog2 up-regulation may orchestrate a two-step mechanism, reducing first the apical area and later down-regulating N-Cadherin expression. A recent study carried out in zebrafish suggested that increased Notch ligand expression during differentiation may recruit Mib1 away from the band 4.1 protein/Ezrin/Radixin/Moesin domain (FERM) protein Erythrocyte membrane protein band 4.1-like 5 (Epb41l5), allowing the latter to accumulate and reduce Cadherin apical levels [49]. This may provide prospective neurons at the verge of differentiation with an additional layer of regulation to dismantle adherens junctions. Whether a similar mechanism also takes place in higher vertebrates will need to be further investigated. We propose that the constriction of the apical domain is necessary to restrict low N-Cadherin levels to small fractions of the junctional network, failure to do so resulting in breaches in the ventricular wall. The actual delamination process was shown in the NT to involve the abscission of the apical cell membrane, leaving an apical remnant at the surface [1]. This event could act as a final stitching step, ensuring the continuity of the ventricular network.

While Notch activity needs to be maintained in prospective neurons, its role during this transition period will need to be clarified. Following Notch blockade, the sequence of events leading to delamination is no longer respected, leading to the appearance of differentiation markers in cells displaying large apical domains. Remarkably, forcing apical constriction through Shroom3 overexpression is sufficient to allow proper delamination. The Notch pathway itself is unlikely to regulate a Shroom-like activity, as forced expression of Neurog2 led to apical constriction from 12 hae onwards (Fig 2A), while Notch levels remained unaffected up to 24 hae (S3C Fig). However, it may play a permissive role in prospective neurons by maintaining epithelial features. Shroom3 was shown to be recruited to adherens junctions by the p120-catenin protein (Adherens junction protein p120) [50]. Thus, Shroom activity may only be compatible with the presence of the apical junctional complex and be lost as neuronal differentiation takes place.

We have investigated the mechanisms that regulate the level of Notch activity during these steps. We confirm previous results showing that Dll1 is required for differentiation [14]. However, in contrast to what had been proposed based on in vitro assays [14], we provide here strong evidence that Dll1 reduces Notch activity cell autonomously through a *Cis*-inhibition mechanism. The ability of Notch ligands to *Cis*-inhibit the receptors has been previously documented in *Drosophila* (for review see [20]). However, in vivo evidence for *Cis*-inhibition in vertebrates is still scarce. Dll3 acts exclusively as a *Cis*-inhibitor [23] and was shown to play a role in T-cell development [26]. But all other Notch ligands can carry out both *Trans*- and *Cis*-activities, making loss-of-function experiments extremely difficult to interpret. In this study, we have taken advantage of the ubiquitin ligase Mib1’s ability to promote *Trans*-activation, to distinguish between *Trans*- and *Cis*- phenomena. Mib1 promotes *Trans*-activation and blocks the ability of Dll1 to induce differentiation. Conversely, blocking Mib1 activity strongly reduces Notch activity cell autonomously and accelerates differentiation, providing a strong demonstration that *Cis*-inhibition takes place in the vertebrate nervous system.

In addition, our study reveals that Mib1 controls a dynamic switch between an initial, transient *Trans*-activating role and a subsequent *Cis*-inhibitory activity of Dll1 during the neural differentiation process (Fig 6). The timing of this switch is not only important for the differentiating cell but also non–cell autonomously, to maintain tissue architecture during the delamination process. The mechanisms underlying both Dll1 *Cis*-inhibition and its blockade by Mib1 will need to be carefully investigated in the future. *Cis*-inhibition was proposed to rely either on the degradation of the Notch receptor or on its titration [20, 51]. Mib1, as it promotes *Trans*-activation, induces the endocytosis of Dll1 and may therefore reduce the amount of Dll1 available for *Cis*-inhibition. It is also possible that Mib1 enhances the affinity of Dll1 for Notch receptors located in *Trans*. Finally, the mechanisms that allow *Cis*-inhibition to take place and the cell to eventually differentiate will need to be addressed. This is likely to result from an increase in the Dll1/Mib1 ratio at the cell membrane. Dll1 is an early target of Neurog2 [35] and was described to increase progressively during differentiation [3]. Dll1 may therefore be progressively induced by Neurog2 and eventually reach a threshold sufficient to carry out *Cis*-inhibition. Consistent with this, co-expression of Dll1 with Neurog2 increases the effect of either construct on differentiation and induces breaches in the ventricular surface (S6D and S6E Fig), whereas shRNA against Dll1 (shDll1) reduces the effect of Neurog2 expression (S6F Fig). On the other hand, Mib1 levels can be decreased through microRNA targeting of its messenger or protein degradation [52, 53].

The developing NT displays the fascinating capacity to transit from a tightly packed epithelium to a meshwork of differentiated neurons and glia while maintaining a cohesive luminal surface. By studying early steps of neurogenesis, we show that prospective neurons maintain epithelial features until their apical endfoot has sufficiently shrunk and can extract itself harmlessly from the ventricular surface. It will be interesting in the future to investigate whether more complex mechanisms are involved as the progenitor pool is used up and ependymal cells are faced with the difficult task of tiling the ventricular system and spinal cord central canal.

## Materials and methods

### Ethics statement

All animal experiments, breeding, and care was compliant with the UK Animals (Scientific Procedures) Act 1986 and was authorized under a project license approved by the Roslin Institute Animal Welfare and Ethical Review Body and the UK Home Office.

Experiments performed with non-hatched avian embryos in the first two thirds of embryonic development time are not considered animal experiments according to the Directive 2010/63/EU.

### Embryos

JA57 chicken fertilized eggs were provided by EARL Morizeau (8 rue du Moulin, 28190 Dangers, France). They were incubated at 38 °C in a Sanyo MIR-253 incubator for the appropriate time.

### Production of the Hes5-VNP transgenic chicken line

The Hes5-VNP-NLS-PEST (Hes5-VNP) reporter transgene [28] was cloned into a lentiviral vector in reverse orientation to prevent the polyA sequence of the transgene from negatively affecting lentiviral packaging efficiency. Transgenic chicken production was carried out by injection of packaged pseudovirus generated from the Hes5-VNP lentiviral vector into blastoderm-stage chicken embryos in new laid eggs. Injected embryos were cultured to hatch and of six chicks, one male was shown to have the transgene present in blood DNA and, at sexual maturity, in semen DNA. The chimeric male (NOR4-21) was bred with stock hens and two transgenic G_1_ male offspring were identified at hatch (NOR4-21:92 and:108). The position of the transgene insert sites in the chicken genome was determined by nested primer amplification of the insert site followed by sequencing, for both G_1_ cockerels. Both carried a single transgene insert site in noncoding regions of the genome. A homozygous transgenic line was established from NOR4-21:92 to provide embryos homozygous for the Hes5-VNP transgene.

### In ovo electroporation and plasmids

Electroporation in the chick NT was performed at E2 or E3 by applying 5 pulses of 50 ms at 25 V with 100 ms in between. For mosaic transfection analysis (Fig 5C, S5B and S5D Fig), lower voltage (3 pulses of 50 ms at 15 V with 950 ms in between) were applied to obtain isolated cells.

The following constructs have been previously described: pCX-EGFP-ZO1 [54], a gift from F. Matsuzaki; pCIG [55]; pCAGGS-ΔMaml1-EGFP [41], a gift from C. Marcelle; pRFP-RNAiC-cDll1-A and pRFP-RNAi-cDll1-B [41], a gift from C. Marcelle, were electroporated together and pRFP-RNAiC [56] was used as a control; pCA-Flag-Shroom3-Full and pCA-EGFP-HA-RII-C1 [40], a gift from M. Takeichi; pCAGGS-cMib1 [27]; pCAGGS-NICD was purchased from Addgene [57].

The following constructs were generated for this study: pCAGGS-Ngn2 was obtained by removing the IRES-GFP fragment from pCIG-Ngn2 [58], a gift from K. Storey. The chick version of Dll1 (cDll1) was cloned and inserted into pCAGGS and pCAGGS-IRES-H2B-Cherry. To generate a membrane-tethered version of Mib1 (pCAGGS-mbMib1), a myristoylation membrane localization sequence (MGCIKSKEDKGPAM from c-Yes kinase [59]) was inserted N-terminally upstream of Mib1, to not interfere with the C-terminal ring finger enzymatic domain of Mib1. For the dominant negative Mib1 (ΔMib1) [60], a version lacking the ring finger domain (aa 1–767) was amplified from the cMib1 and inserted into pCAGGS and pCAGGS-IRES-H2B-Cherry [27]. Other plasmids used are: pCX-EGFP (0.5 μg/μL), pCX-H2B-EGFP (0.5 μg/μL), pCX-iRFP-ZO1 (0.2 μg/μL), and pCAGGS-TetOn-IRES-H2B-iRFP (0.2 μg/μL). All plasmids were used at 1 μg/μL except where otherwise mentioned.

### FlashTagging

FlashTagging procedures were adapted from [31]. CellTrace CFSE (Life Technologies, #C34554) was injected at 0.5 mM concentration into E2(HH12) or E2.75 chick NT. Embryos were incubated at 38 °C for the appropriate time until dissection. EdU was deposited at 4 h intervals as described below and schematized in Fig 1C and S2B Fig.

### EdU labeling

Proliferating cells in the NT were labeled by in ovo incorporation of 5-ethynyl-2′-deoxyuridine (EdU). One hundred microliters of a 100 μM solution of EdU diluted in PBS was deposited on the embryo. Embryos were incubated for 1 h (S3B Fig) or more for cumulative EdU labeling (Fig 1C, S2C–S2E Fig), then dissected and fixed as described above. Immunodetection of EdU incorporated cells was carried out on cryostat sections using the Click-iT EdU imaging kit (Invitrogen).

### Immunohistochemistry

Chick embryos were fixed for 1 h in ice-cold 4% formaldehyde/PBS and rinsed 3 times in PBS. For cryosections, they were equilibrated at 4 °C in PB/15% sucrose and embedded in PB/15% sucrose/7.5% gelatin before sectioning. Before immunostaining, cryosections were equilibrated at room temperature, degelatinized in PBS at 37 °C 3 times 5 min, before a 30-min blocking step in PBS-0.1%Triton/10% fetal calf serum (FCS). Slides were then incubated with the primary antibodies diluted in the blocking solution at 4 °C overnight. The following day, slides were washed 3 times 5 min in PBS-0.1%Triton, incubated 2 h with the adequate secondary antibodies at room temperature, washed again 3 times, and mounted with DAPI containing Vectashield (Vector Labs).

For en face views, fixed embryos were cut along their midline and bathed 1 h in blocking solution (PBS-0.3%Triton/10%FCS), followed by overnight incubation at 4 °C with the primary antibodies diluted in the blocking solution. The next day, embryos were washed 4–5 times with PBS-0.3%Triton, incubated overnight at 4 °C with the secondary antibodies, washed again 3 times 10 min in PBS-0.3%Triton and flat-mounted (apical side facing the coverslip) with DAPI containing Vectashield.

Primary antibodies used are: chicken anti-GFP (Aves Lab, 1:800); mouse anti-HuCD (clone 16A11, Life Technologies, 1:50); guinea-pig anti-Neurog2 (a gift from B. Novitch [61] 1:32,000); rabbit anti-phospho-Histone H3 (Millipore, 1:250); rabbit anti-Pax6 (Millipore, 1:500); mouse anti-N-Cadherin (clone GC-4, Sigma Aldrich, 1:100) (BD Biosciences, 1:250); mouse anti-βIII-tubulin (clone Tuj1; Covance, 1:500); rabbit anti-Par3 (Millipore, 1:1,000); mouse anti-ZO1 (clone 1A12, ThermoFischer, 1:100); goat anti-Sox2 (clone Y-17, Santa Cruz, 1:100). Secondary antibodies coupled to Alexa Fluor 488, Cy3, or Alexa Fluor 649 were obtained from Jackson laboratories.

### In situ hybridization

In situ hybridization on gelatin mounted cryosections was performed as previously described [62]. All of the probes were synthesized using a DIG RNA labeling kit (Roche) as specified by the manufacturer. Antisense probes were prepared from the following linearized plasmids: cHes5.1 (a gift from D. Henrique), cHes1 (a gift from S. Bel-Vialar), and cDll1 (a gift from Olivier Pourquié). To generate hΔMaml1, cMib1, mShroom3, and mRII-C1 antisense probes, primers containing T3 and T7 overhangs were used to PCR amplify a region from the corresponding expression plasmids. The purified amplicon was then used as the template for antisense probe synthesis using T3 or T7 RNA polymerase.

Gelatin-mounted cryosections from overnight-fixed tissue were equilibrated at room temperature and degelatinized in PBS at 37 °C 3 times 5 min. Slides were treated 20 min in RIPA buffer (150 mM NaCl, 1% NP-40, 0.5% Na deoxycholate, 0.1% SDS, 1 mM EDTA, 50 mM Tris pH 8.0), postfixed in 4% paraformaldehyde/PBS for 10 min, and washed 3 times 5 min with PBS. The slides were then transferred in Triethanolamine buffer (100 mM triethanolamine, acetic acid 0.25% pH 8.0) for 15 min and washed 3 times 5 min in PBS. Slides were prehybridized during 1 h with 500 μL of hybridization solution (50% formamide, 5X SSC, 5X Denhardt’s, 500 μg/mL herring sperm DNA, 250 μg/mL yeast RNA) and hybridized overnight at 70 °C with the same solution in the presence of the heat-denatured DIG-labeled RNA probes. The following day, slides were placed in post-hybridization solution (50% Formamid; 2X SSC; 0.1% Tween20) at 70 °C, then washed in 0.2X SSC for 30 min at 70 °C and finally in 0.2X SSC at RT for 5 min. Slides were washed with buffer 1 (100 mM maleic acid, pH 7.5, 150 mM NaCl, 0.05% Tween 20) during 20 min at room temperature, blocked for 30 min in buffer 2 (buffer 1/10% FCS), followed by overnight incubation at 4 °C with the anti-DIG antibody (Roche) diluted 1:2,000 in buffer 2. The following day, slides were washed 3 times 5 min with buffer 1 and equilibrated for 30 min in buffer 3 (100 mM Tris pH 9.5, 100 mM NaCl, 50 mM MgCl_2_). The signal was visualized by a color reaction using 500 μL of BM-Purple (Roche). The color reaction was allowed to develop in the dark at room temperature during 30 min–4 h and was stopped with PBS-0.1% Tween20.

### Image acquisition and processing

Optical sections of fixed samples (en face views from half embryos or transverse views from cryosections) after immunofluorescence were obtained on a confocal microscope (model SP5; Leica) using 20× and 63× (Plan Neofluar NA 1.3 oil immersion) objectives and Leica LAS software. For image processing and data analysis, we used the ImageJ and FIJI software [63, 64]. Images were finally subjected to brightness and contrast adjustments to equilibrate channel intensities and background using ImageJ and FIJI software.

### Image quantifications

#### Hes5-VNP signal intensity measurement and color code

Hes5-VNP signal intensity was obtained by measuring the VNP fluorescence average pixel intensity of a nuclei area defined using the DAPI channel. As Notch blockade with DAPT treatment reduced the Hes5-VNP signal intensity down to the level measured in neurons (S1C Fig), we considered the latter as background. Therefore, for each experiment, the VNP intensity measured in neurons was averaged and subtracted from all values, which were then all normalized to the average value measured in progenitors. Importantly, for each experimental condition and its control, all pictures were taken at the confocal microscope using identical parameters and during a unique session, except for clonal analyses (Fig 5C and S5D Fig). In this last case, pictures taken during different confocal sessions were normalized between them using the mean of VNP fluorescence average pixel intensity (minus background) of HuCD^+^ nuclei of the non-electroporated side as reference. Quantifications in Fig 4D (two first columns) and S5C Fig; S3B and S6A Figs (two first columns) come from the same data sets. The color coded map of Hes5-VNP signaling (Fig 1B) was obtained using two consecutive macros in FIJI software. Briefly, the VNP fluorescence average pixel intensity (minus background) of a nucleus area manually defined using the DAPI channel and its x–y position and shape descriptors were recorded in a FIJI Results Table using a first macro. A second macro was then used to generate the color coded map, in which each nucleus was redrawn as an ellipse using the recorded x, y, and shape descriptor values and assigned a given color based on its VNP fluorescence intensity.

#### Apical area and N-Cadherin intensity ratio

The apical area ratio was obtained by dividing the apical area of a transfected cell by the mean apical area of four of its non-transfected close neighbors (spaced by one cell row from the transfected cell). The N-Cadherin intensity ratio was obtained by dividing the average pixel intensity (minus background) measured within the apical circumference of a transfected cell by that of four of its close non-transfected neighbors.

#### Differentiation and proliferation rate

The proliferation and differentiation rates were obtained by dividing the number of transfected EdU^+^ and HuCD^+^ cells by the total number of transfected cells. As progenitors differentiate much earlier in the ventrally located motor neuron domain, we concentrated our analysis on the dorsal two thirds of the NT in order to reason on a more homogenous progenitor population. The differentiation rate in neighboring cells (Fig 4C) was obtained by dividing the number of non-transfected HuCD^+^ cells adjacent to a transfected HuCD^+^ cell by the total number of non-transfected cells adjacent to the transfected HuCD^+^ cell.

### Statistical analyses

The number of embryos and analyzed cells or sections are indicated in the figure legends. All data processing and statistical analyses were performed using Excel and GraphPad Prism softwares. For data following a normal distribution, significance was assessed using either a Student *t* test (S1B-Right, S2E, S2F, S3A, S3B, S3D, S3F, S5A, S5B and S6C Figs) to compare the mean of two groups or one-way ANOVA (Figs 2A–2C, 4C, 4E, 5B and 5D, S2D, S4D, S4E, S6A, S6B, S6D and S6F Figs) with Tukey correction to compare the mean of three or more groups. Data represent mean + SEM, ns, *p* > 0.05; *p < 0.05; **p < 0.01, ***p < 0.001. For the analysis of Hes5-VNP intensity distributions, significance was assessed using a Mann-Whitney *U* test (Fig 5C, S1B-Left, S3C, S3E, S5C and S5D Figs) to compare the median of two groups or a Kruskal-Wallis test (Figs 1C, 1D, 4B, 4D and 5A, S1C Fig) with Dunn’s correction to compare the median of three or more groups. ns, *p* > 0.05; *p < 0.05; **p < 0.01, ***p < 0.001.

### DAPT NT culture

A trunk explant spanning the brachial to lumbar region was dissected from E3 Hes5-VNP embryos and grown in culture medium (F12/Penicillin Streptomycin/Sodium pyruvate 1 mM) for 8 h at 38.5 °C. We added to the culture medium either DAPT (N-(3,5-difluorophenylacetyl-L-alanyl)-S-phenylglycine t-ButylEster [InSolution γ-Secretase Inhibitor IX; Calbiochem] at a final concentration of 10 μM dissolved in DMSO) or DMSO alone at the indicated time (see schematic in S1C Fig). At the end of the culture period, embryos were fixed as described above and processed for immunohistochemistry.

## Supporting information

S1 Fig Hes5-VNP Notch reporter chicken line. **(A)** Transverse sections of the NT of the Hes5-VNP transgenic line at E4. Adjacent sections were used to visualize the Hes5-VNP signal revealed by anti-Venus immunostaining (green), with *cHes5*.*1* and *cHes1* expression detected by in situ hybridization. **(B)** Left: Transverse section of the NT of the Hes5-VNP transgenic line transfected at E2 with NICD, harvested at E3 and immunostained for Venus (green) and HuCD (blue) to label neurons. Transfection is reported by H2B-iRFP expression (red). Middle: Quantification of the Hes5-VNP intensity measured in HuCD^−^ cells transfected at E2 in control (non-electroporated side) and NICD conditions and harvested 24 hae. Data represent fold change compared to control, calculated from 105 cells collected from five embryos for each group. ***p < 0.001 (Mann-Whitney *U* test). Right: Quantification of the differentiation rate (number of HuCD^+^ cells on total transfected cells) in control and NICD conditions 24 and 48 hae. Data represent mean + SEM. For 24 hae, *n* = 14 (4 embryos), 13 (4 embryos) for control and NICD, respectively. For 48 hae, *n* = 14 (3 embryos), 15 (4 embryos) sections for control and NICD, respectively. ***p < 0.001 (Student *t* test). **(C)** Left: Transverse sections of the NT of the Hes5-VNP transgenic line at E3 treated with DMSO or DAPT during the indicated times. The time course of the protocol is schematized below. All embryos were cultured for 8 h; DAPT (10 μM) was added to the culture medium at the indicated time. Right: Quantification of the Hes5-VNP signal intensity fold change in HuCD^−^ cells, in DMSO and DAPT treated embryos. At least 100 cells were measured from two embryos for each experimental group. ***p < 0.001 (Kruskal-Wallis test). Underlying data are provided in S1 Data. Scale bar represents 50 μm. DAPT, N-(3,5-difluorophenylacetyl-L-alanyl)-S-phenylglycine t-ButylEster; E, embryonic day; H2B, Histone 2B; hae, hour after electroporation; Hes5, Hairy and Enhancer of Split 5; HuCD neuron-specific RNA-binding proteins HuC and HuD; iRFP, infrared fluorescent protein; NICD, Notch intracellular domain; NT, neural tube; VNP, Venus-NLS-PEST.
(TIF)

S2 Fig Characterization of prospective neurons. (A) Transverse sections of the NT injected with FT at E2.75, harvested at the indicated time points, and immunostained with phospho-Histone H3. (B) Schematic outline of the experimental protocol represented in (C). All embryos were injected with FT at the same time; EdU was administrated 3 h after FT, then every 4 h, and harvested at the indicated time. (C) Transverse sections of the NT injected with FT at E2.75, incubated with continuous EdU, and harvested at the indicated time points. FT is shown in green; red stainings reveal EdU (middle row) or the neuronal marker HuCD (bottom row). Arrowheads indicate double FT^+^/HuCD^+^ cells. (D) Quantification of the proliferation rate (number of EdU^+^ cells on total FT^+^ cells) and differentiation rate (number of HuCD^+^ cells on total FT^+^ cells) in embryos injected with FT at E2(HH12) or at E2.75 and analyzed at the indicated time points. ns, *p* > 0.05 (one-way ANOVA). (E) Left: Transverse sections of the dorsal NT incubated with continuous EdU (red) and stained with Neurog2 (green). Right: Quantification of the proliferation rate (proportion of EdU^+^ cells in Neurog2^−^ and Neurog2^+^ populations). Data represent mean + SEM. *n* = 10 collected from five embryos were analyzed. ***p < 0.001 (Student *t* test). (F) Left: Transverse sections of the dorsal NT at E4 immunostained for Neurog2 (green) and HuCD (red). Right: Quantification of the differentiation rate (number of HuCD^+^ cells on Neurog2^Low^ and Neurog2^High^ cells). Data represent mean + SEM. *n* = 9 sections collected from six embryos were analyzed. *p < 0.05 (Student *t* test). Underlying data are provided in S1 Data. Scale bar represents 25 μm. E, embryonic day; EdU, 5-ethynyl-2′-deoxyuridine; FT, FlashTag; HH12, Hamburger-Hamilton stage 12; HuCD, neuron-specific RNA-binding proteins HuC and HuD; Neurog2, Neurogenin 2; ns, nonsignificant; NT, neural tube.
(TIF)

S3 Fig Effects of Neurog2 and ΔMaml1 overexpression on Notch signaling and neurogenesis. **(A)** Left: Transverse sections of the NT transfected at E2 with Neurog2, harvested at E3 and immunostained for Pax6 (red). Transfection is reported by GFP expression. Right: Quantification of the number of Pax6^+^ cells on total transfected cells. Note that the quantification was performed on the Pax6 positive domain (inside the white dotted lines). Electroporation with Neurog2 results in efficient knockdown of Pax6. Data represent mean + SEM. *n* = 8 and 6 sections collected from three embryos were analyzed for control and Neurog2, respectively. ***p < 0.001 (Student *t* test). **(B)** Left: Transverse sections of the NT transfected at E2 with the indicated constructs and harvested at E3. Transfection is reported by GFP expression. S-phase proliferating cells were labeled by EdU after a 1 h pulse (red). Right: Quantification of the proliferation rate (number of EdU^+^ cells on total transfected cells) 24 hae. Data represent mean + SEM. *n* = 10 (4 embryos) and 12 (4 embryos) sections were analyzed for control and Neurog2, respectively. ***p < 0.001 (Student *t* test). **(C, E)** Left: Transverse sections of the dorsal NT in the Hes5-VNP transgenic line transfected at E2 with the indicated constructs harvested at E3 and immunostained for Venus (green). Transfection is reported by H2B-iRFP expression (red). Right: Quantification of the Hes5-VNP signal intensity in HuCD^−^ cells in control (non-electroporated side), **(C)** Neurog2, and **(E)** ΔMaml1 conditions. A minimum of *n* = 84 cells **(C)** or *n* = 51 cells **(E)** collected from four embryos were analyzed for each group. ns, *p* > 0.05; ***p < 0.001 (Mann-Whitney *U* test). **(D, F)** Left: Transverse sections of the NT transfected at E2 with the indicated constructs, harvested 24 hae or 48 hae and immunostained for HuCD (red) to label neurons. Transfection is reported by GFP expression. Right: Quantification of the differentiation rate (number of HuCD^+^ cells on total transfected cells) 24 hae and 48 hae. Data represent mean + SEM. **(D)** For 24 hae, *n* = 13 (9 embryos) and 13 (6 embryos) were analyzed for control and Neurog2, respectively. For 48 hae, *n* = 13 (6 embryos) and 15 (6 embryos) sections were analyzed for control and Neurog2, respectively. **(F)** For 24 hae, *n* = 14 (9 embryos) and 15 (9 embryos) sections were analyzed for control and ΔMaml1, respectively. For 48 hae, *n* = 10 (6 embryos) and 17 (6 embryos) sections were analyzed for control and ΔMaml1, respectively. ***p < 0.001 (Student *t* test). Underlying data are provided in S1 Data. **(G)** Transverse sections of the NT transfected at E2 with ΔMaml1 and harvested at E3. Adjacent sections were used to visualize electroporation efficiency with GFP expression and to reveal *hΔMaml1* expression by in situ hybridization. + indicates the electroporated side of the NT. Scale bar represents 50 μm **(A–B, D, F–G)** or 25 μm **(C, E)**. ΔMaml1, dominant-negative Mastermind-like 1; E, embryonic day; EdU, 5-ethynyl-2′-deoxyuridine; GFP, green fluorescent protein; H2B-iRFP, Histone 2B fused to infrared fluorescent protein; hae, hour after electroporation; Hes5, Hairy and Enhancer of Split 5; HuCD, neuron-specific RNA-binding proteins HuC and HuD; iRFP, infrared fluorescent protein; Neurog2, Neurogenin 2; ns, nonsignificant; NT, neural tube; Pax6, Paired box gene 6; VNP, Venus-NLS-PEST.
(TIF)

S4 Fig Effects of Shroom3 and RII-C1 overexpression on neuron delamination. **(A)** Transverse sections of the NT transfected at E2 with the indicated constructs and harvested at E3. Adjacent sections were used to visualize electroporation efficiency with GFP expression and to reveal *mShroom3* or *mRII-C1* expression by in situ hybridization. Scale bar represents 50 μm. **(B)** Transverse views of the NT transfected at E2 with the indicated constructs, harvested at E3 and immunostained for the apical markers Par3 and ZO1. + indicates the transfected side of the NT. Scale bar represents 25 μm. **(C)** Apical views of the NT at E2 transfected with ZO1-iRFP (green) along with the indicated constructs, harvested 18 hae and immunostained for N-Cadherin. The boxed areas indicate the cell of interest. Scale bar represents 2 μm. **(D, E)** Quantification of the apical area ratio (ratio of the area of a transfected cell on the mean area of four of its close non-transfected neighbors) and N-Cadherin level ratio (ratio of the average pixel intensity within the apical circumference of one transfected cell corrected by the background versus the mean of average pixel intensity of four of its close non-transfected neighbors). Data represent mean + SEM. ns, *p* > 0.05; *p < 0.05; **p < 0.01; ***p < 0.001 (one-way ANOVA). Underlying data are provided in S1 Data. E, embryonic day; GFP, green fluorescent protein; hae, hour after electroporation; iRFP, infrared fluorescent protein; ns, nonsignificant; NT, neural tube; Par3, Partition defective protein 3; RII-C1, Shroom3 binding site on ROCK2; Shroom3, shroom family member 3; ZO1, zonula occludens 1.
(TIF)

S5 Fig Dll1 levels control neurogenesis through the regulation of Notch activity. **(A, B)** Left: Transverse sections of the NT transfected at E2 **(A)** or E3 **(B)** with the indicated constructs, harvested at E4 **(A)** or E5 **(B)** and immunostained for HuCD (green) to label neurons. Transfection is reported by H2B-Cherry or RFP expression. In **(B)**, electroporation was performed at low voltage (15 V) to obtain mosaic transfections. Right: Quantification of the differentiation rate (number of HuCD^+^ cells on total transfected cells). Data represent mean + SEM. **(A)** *n* = 12 and 15 sections collected from six embryos for each experimental group were analyzed for control and Dll1, respectively. **(B)** *n* = 36 sections (6 embryos) and 40 sections (8 embryos) were analyzed for control and shDll1, respectively. ***p < 0.001 (Student *t* test). **(C, D)** Left: Transverse section of the NT of the Hes5-VNP transgenic line transfected at E2 with Dll1 **(C)** or at E3 with shDll1 **(D)** constructs and their respective controls, harvested 24 hae and immunostained for Venus (green) and HuCD (blue) to label neurons. Transfection is reported by H2B-iRFP **(C)** or RFP **(D)** expression (red). Right: Quantification of Hes5-VNP intensity in HuCD^−^ cells transfected with the indicated constructs at E2 **(C)** or E3 **(D)** with a normal **(C)** or low voltage **(D)** condition and harvested 24 hae. Data represent fold change compared to control. **(C)** *n* = 58 and 59 cells collected from six embryos for each experimental group were analyzed for control and Dll1, respectively. **(D)** *n* = 35 and 42 cells collected from 11 embryos for each experimental group were analyzed for control and shDll1, respectively. **p < 0.01; ***p < 0.001 (Mann-Whitney *U* test). Underlying data are provided in S1 Data. **(E)** Transverse sections of the NT transfected at E2 with the indicated constructs and harvested at E3. Adjacent sections were used to visualize electroporation efficiency with H2B-GFP or H2B-Cherry expression and to reveal *cDll1* or *cMib1* expression by in situ hybridization. + indicates the electroporated side of the NT. Scale bar represents 50 μm. Dll1, Delta-like 1; E, embryonic day; hae, hour after electroporation; H2B-Cherry, Histone 2B fused to Cherry; H2B-GFP, Histone 2B fused to GFP; Hes5, Hairy and Enhancer of Split 5; HuCD, neuron-specific RNA-binding proteins HuC and HuD; iRFP, infrared fluorescent protein; NT, neural tube; RFP, red fluorescent protein; shDll1, shRNA against Dll1; VNP, Venus-NLS-PEST.
(TIF)

S6 Fig Synergistic effects of Neurog2 and Dll1 forced expression on differentiation and neuroepithelial integrity. **(A)** Quantification of the proliferation rate (number of EdU^+^ cells on total transfected cells) 24 hae. Data represent mean + SEM. *n* = 10, 12, 23, and 20 sections collected from four embryos for each experimental group were analyzed for control, Neurog2, Neurog2+NICD, and NICD, respectively. **(B)** Quantification of the differentiation rate (number of HuCD^+^ cells on total transfected cells) 48 hae. Data represent mean + SEM. *n* = 9, 9, 9, and 7 sections collected from three embryos for each experimental group were analyzed for control, Neurog2, Neurog2+NICD, and NICD, respectively. ns, *p* > 0.05; **p < 0.01; ***p < 0.001 (one-way ANOVA). **(C, D)** Left: Transverse sections of the NT transfected at E2 with the indicated constructs, harvested at E3 and immunostained for HuCD (green) to label neurons. Transfection is reported by H2B-Cherry expression (red). Right: Quantification of the differentiation rate (number of HuCD^**+**^ cells on total transfected cells). Data represent mean + SEM. **(C)** *n* = 10 and 12 sections collected from four embryos for each experimental group were analyzed for control and Dll1, respectively. **(D)** *n* = 8, 9, and 15 sections collected from six embryos for each experimental group were analyzed for control, Neurog2, and Neurog2+Dll1, respectively. **p < 0.01; ***p < 0.001 (one-way ANOVA). **(E)** Transverse sections of the NT transfected at E2 with the indicated constructs, harvested at E3, and immunostained for N-Cadherin (red). Transfection is reported by H2B-Cherry expression (green). N-cadherin is down-regulated on the electroporated side upon double Neurog2+Dll1 expression; asterisk indicates breach to the ventricular wall. **(F)** Left: Transverse sections of the NT transfected at E2 with the indicated constructs, harvested at E4 and immunostained for HuCD (green) to label neurons. Transfection is reported by RFP expression (red). Right: Quantification of the differentiation rate (number of HuCD^**+**^ cells on total transfected cells). Data represent mean + SEM. **p < 0.01 (one-way ANOVA). *n* = 14, 11, and 16 sections collected from five embryos for each experimental group were analyzed for control, Neurog2, and Neurog2+shDll1, respectively. Underlying data are provided in S1 Data. Scale bar represents 50 μm. Dll1, Delta-like 1; E, embryonic day; H2B-Cherry, Histone 2B fused to Cherry; hae, hour after electroporation; HuCD, neuron-specific RNA-binding proteins HuC and HuD; Neurog2, Neurogenin 2; NICD, Notch intracellular domain; ns, nonsignificant; NT, neural tube; RFP, red fluorescent protein; shDll1, shRNA against Dll1.
(TIF)

S1 Data (XLSX)

## Abbreviations

ΔMaml1: dominant-negative Mastermind-like 1
ΔMib1: dominant-negative Mib1
bHLH: basic helix-loop-helix
cHes5.1: chicken Hairy and Enhancer of Split 5.1
CFSE: carboxyfluorescein succinimidyl ester
DAPT: N-(3,5-difluorophenylacetyl-L-alanyl)-S-phenylglycine t-ButylEster
dI6: dorsal interneuron domain 6
Dll1: Delta-like 1
Dll3: Delta-like 3
E: embryonic day
EdU: 5-ethynyl-2′-deoxyuridine
EP: electroporation
Epb41l5: Erythrocyte membrane protein band 4.1-like 5
FCS: fetal calf serum
FERM: band 4.1 protein/Ezrin/Radixin/Moesin domain
FoxP2/4: Forkhead transcription factors FoxP2 and FoxP4
FT: FlashTag
GFP: green fluorescent protein
hae: hour after electroporation
Hes5: Hairy and Enhancer of Split 5
HuCD: neuron-specific RNA-binding proteins HuC and HuD
H2B: Histone 2B
H2B-Cherry: Histone 2B fused to Cherry
H2B-GFP: Histone 2B fused to GFP
iRFP: infrared fluorescent protein
Mib1: Mindbomb1
mbMib1: Mib1 constitutively tethered to the plasma membrane
Neurog2: Neurogenin 2
NICD: Notch intracellular domain
NT: neural tube
Par3: Partition defective protein 3
Pax6: Paired box gene 6
p120-catenin: Adherens junction protein p120
ROCK: Rho kinase
RII-C1: Shroom3 binding site on ROCK2
shDll1: shRNA against Dll1
shRNA: short hairpin RNA
Shroom3: Shroom family member 3
Sox2: SRY (sex determining region Y) box 2
V1: intermediate ventral interneuron domain 1
VNP: Venus-NLS-PEST
VZ: ventricular zone
ZO1: Zonula Occludens 1
