# Supplementary figures and images for: Mib1 prevents Notch Cis-inhibition to defer differentiation and preserve neuroepithelial integrity during neural delamination
Source: PLoS Biol. 2018 Apr 30;16(4):e2004162. doi: 10.1371/journal.pbio.2004162 (PMC5945229; doi:10.1371/journal.pbio.2004162)

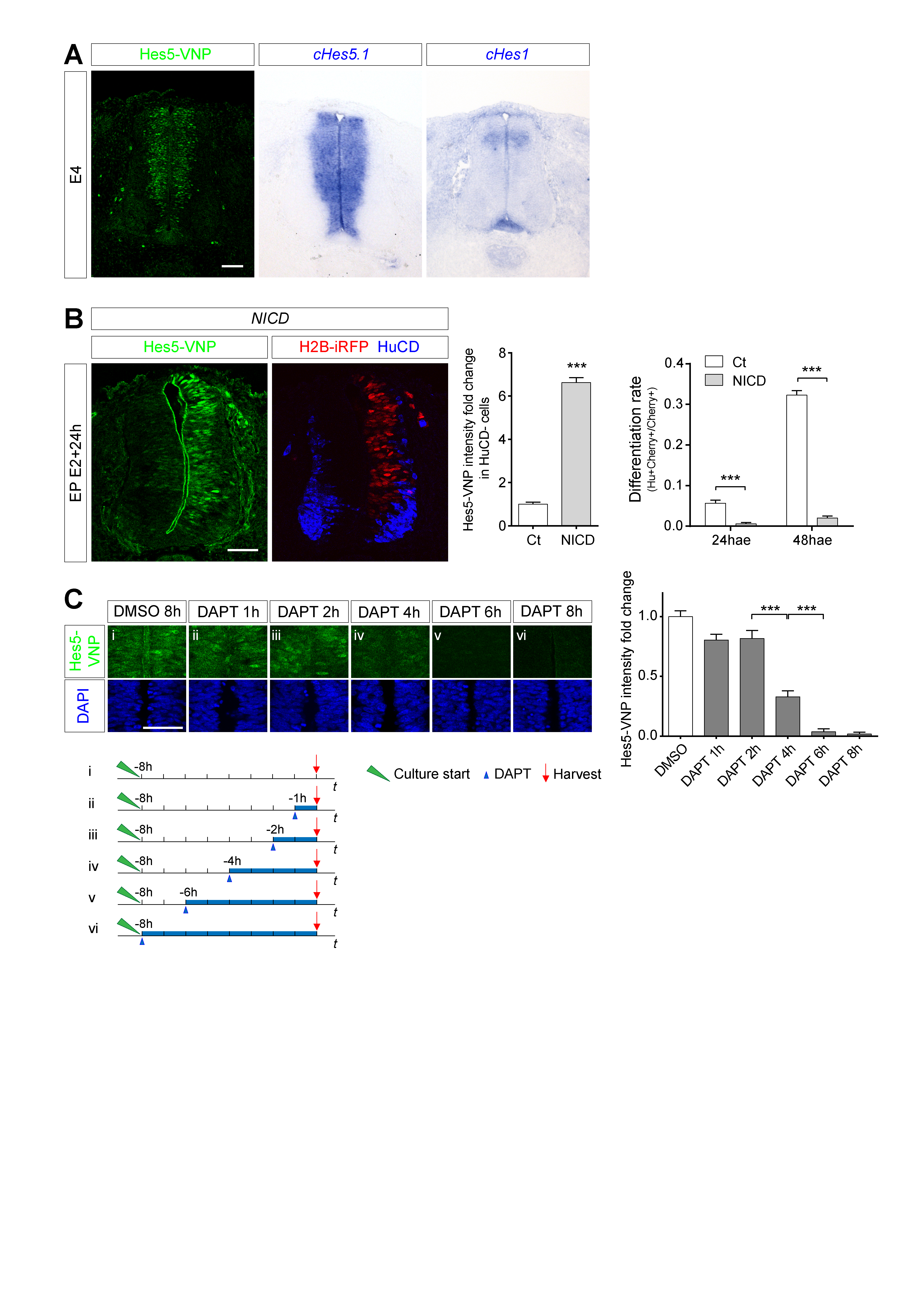

Supplement: S1 Fig — (A) Transverse sections of the NT of the Hes5-VNP transgenic line at E4. Adjacent sections were used to visualize the Hes5-VNP signal revealed by anti-Venus immunostaining (green), with cHes5.1 and cHes1 expression detected by in situ hybridization. (B) Left: Transverse section of the NT of the Hes5-VNP transgenic line transfected at E2 with NICD, harvested at E3 and immunostained for Venus (green) and HuCD (blue) to label neurons. Transfection is reported by H2B-iRFP expression (red). Middle: Quantification of the Hes5-VNP intensity measured in HuCD− cells transfected at E2 in control (non-electroporated side) and NICD conditions and harvested 24 hae. Data represent fold change compared to control, calculated from 105 cells collected from five embryos for each group. ***p < 0.001 (Mann-Whitney U test). Right: Quantification of the differentiation rate (number of HuCD+ cells on total transfected cells) in control and NICD conditions 24 and 48 hae. Data represent mean + SEM. For 24 hae, n = 14 (4 embryos), 13 (4 embryos) for control and NICD, respectively. For 48 hae, n = 14 (3 embryos), 15 (4 embryos) sections for control and NICD, respectively. ***p < 0.001 (Student t test). (C) Left: Transverse sections of the NT of the Hes5-VNP transgenic line at E3 treated with DMSO or DAPT during the indicated times. The time course of the protocol is schematized below. All embryos were cultured for 8 h; DAPT (10 μM) was added to the culture medium at the indicated time. Right: Quantification of the Hes5-VNP signal intensity fold change in HuCD− cells, in DMSO and DAPT treated embryos. At least 100 cells were measured from two embryos for each experimental group. ***p < 0.001 (Kruskal-Wallis test). Underlying data are provided in S1 Data. Scale bar represents 50 μm. DAPT, N-(3,5-difluorophenylacetyl-L-alanyl)-S-phenylglycine t-ButylEster; E, embryonic day; H2B, Histone 2B; hae, hour after electroporation; Hes5, Hairy and Enhancer of Split 5; HuCD neuron-specific RNA-binding pro [file pbio.2004162.s001.tif]

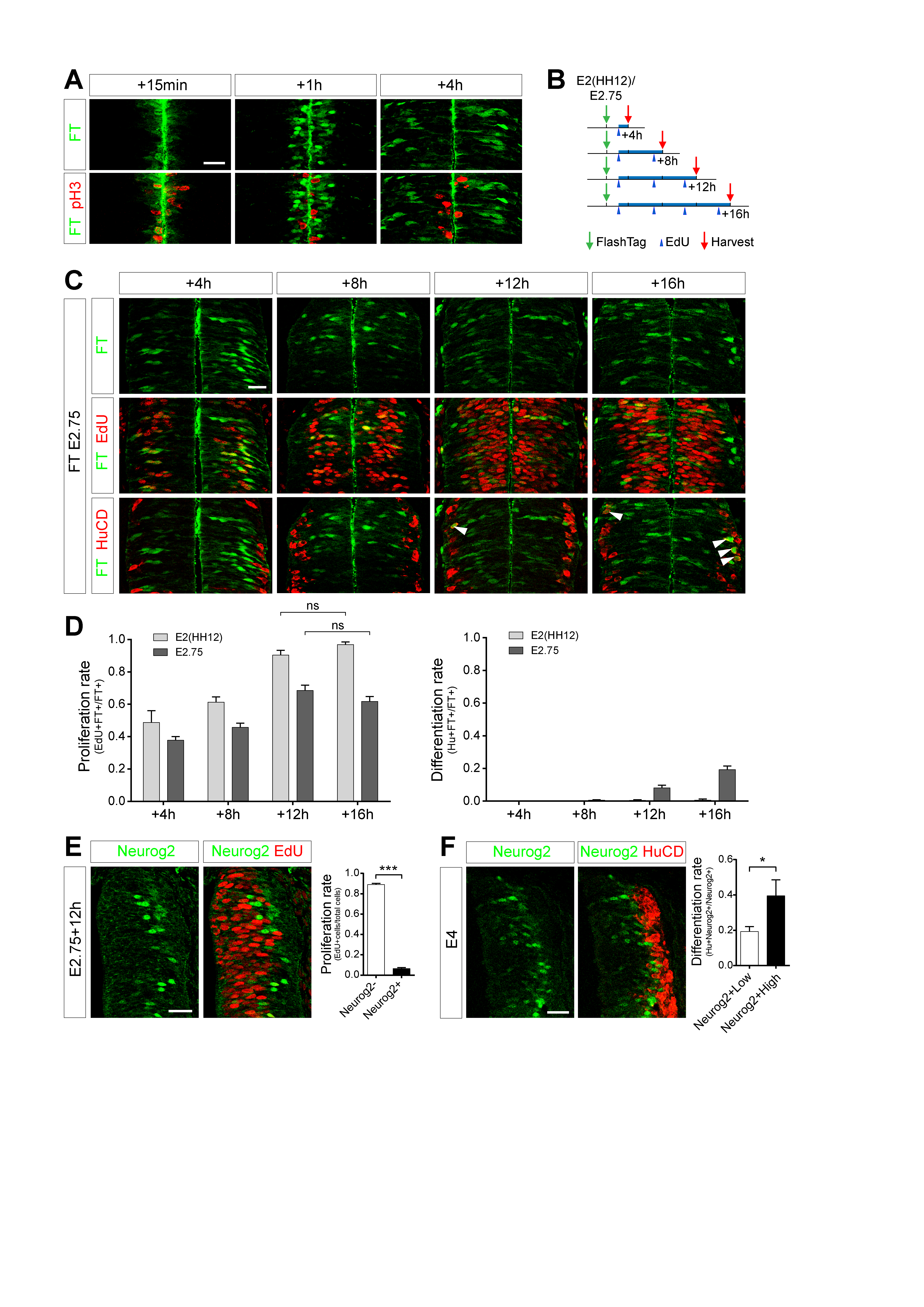

Supplement: S2 Fig — (A) Transverse sections of the NT injected with FT at E2.75, harvested at the indicated time points, and immunostained with phospho-Histone H3. (B) Schematic outline of the experimental protocol represented in (C). All embryos were injected with FT at the same time; EdU was administrated 3 h after FT, then every 4 h, and harvested at the indicated time. (C) Transverse sections of the NT injected with FT at E2.75, incubated with continuous EdU, and harvested at the indicated time points. FT is shown in green; red stainings reveal EdU (middle row) or the neuronal marker HuCD (bottom row). Arrowheads indicate double FT+/HuCD+ cells. (D) Quantification of the proliferation rate (number of EdU+ cells on total FT+ cells) and differentiation rate (number of HuCD+ cells on total FT+ cells) in embryos injected with FT at E2(HH12) or at E2.75 and analyzed at the indicated time points. ns, p > 0.05 (one-way ANOVA). (E) Left: Transverse sections of the dorsal NT incubated with continuous EdU (red) and stained with Neurog2 (green). Right: Quantification of the proliferation rate (proportion of EdU+ cells in Neurog2− and Neurog2+ populations). Data represent mean + SEM. n = 10 collected from five embryos were analyzed. ***p < 0.001 (Student t test). (F) Left: Transverse sections of the dorsal NT at E4 immunostained for Neurog2 (green) and HuCD (red). Right: Quantification of the differentiation rate (number of HuCD+ cells on Neurog2Low and Neurog2High cells). Data represent mean + SEM. n = 9 sections collected from six embryos were analyzed. *p < 0.05 (Student t test). Underlying data are provided in S1 Data. Scale bar represents 25 μm. E, embryonic day; EdU, 5-ethynyl-2′-deoxyuridine; FT, FlashTag; HH12, Hamburger-Hamilton stage 12; HuCD, neuron-specific RNA-binding proteins HuC and HuD; Neurog2, Neurogenin 2; ns, nonsignificant; NT, neural tube. (TIF) [file pbio.2004162.s002.tif]

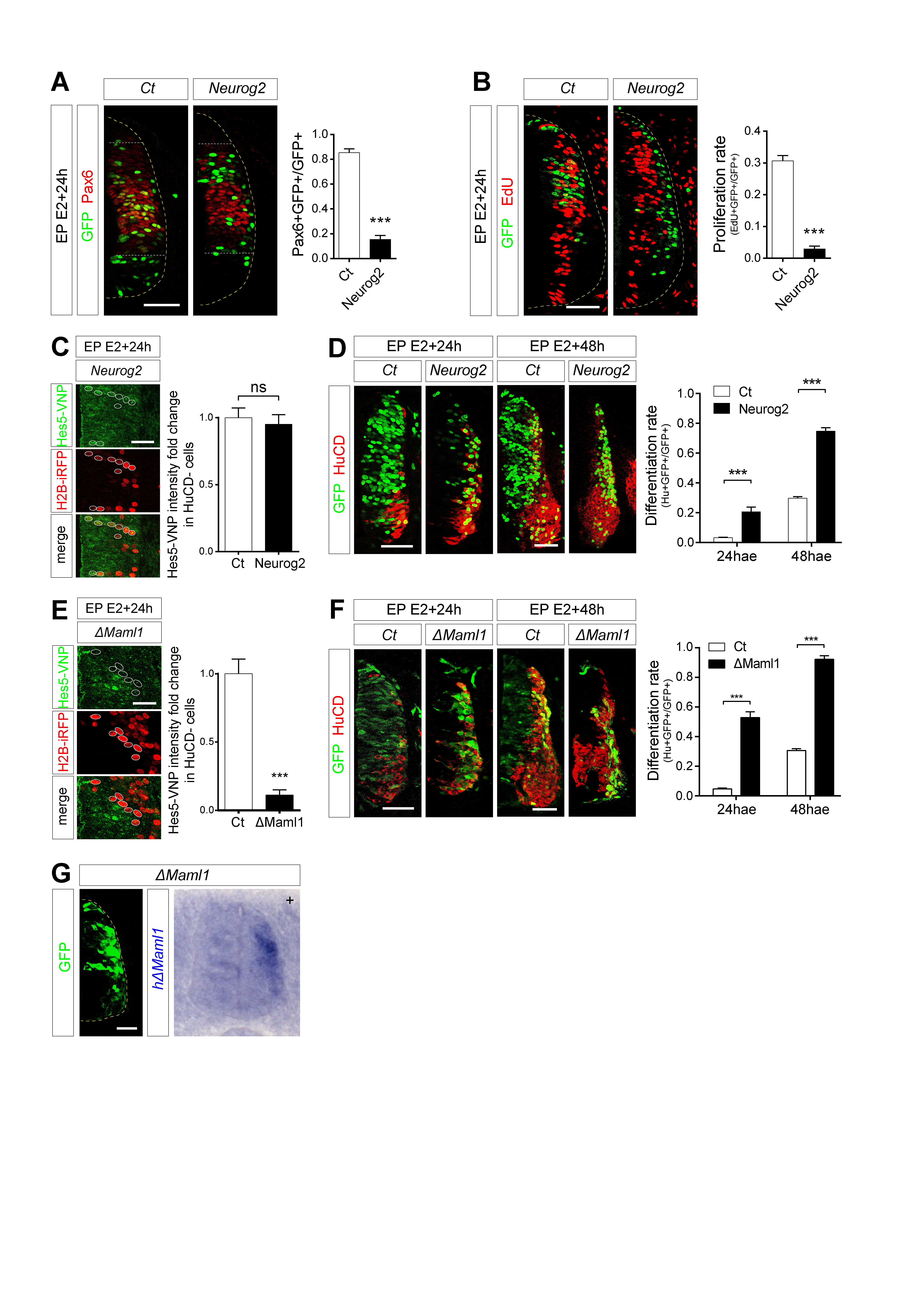

Supplement: S3 Fig — (A) Left: Transverse sections of the NT transfected at E2 with Neurog2, harvested at E3 and immunostained for Pax6 (red). Transfection is reported by GFP expression. Right: Quantification of the number of Pax6+ cells on total transfected cells. Note that the quantification was performed on the Pax6 positive domain (inside the white dotted lines). Electroporation with Neurog2 results in efficient knockdown of Pax6. Data represent mean + SEM. n = 8 and 6 sections collected from three embryos were analyzed for control and Neurog2, respectively. ***p < 0.001 (Student t test). (B) Left: Transverse sections of the NT transfected at E2 with the indicated constructs and harvested at E3. Transfection is reported by GFP expression. S-phase proliferating cells were labeled by EdU after a 1 h pulse (red). Right: Quantification of the proliferation rate (number of EdU+ cells on total transfected cells) 24 hae. Data represent mean + SEM. n = 10 (4 embryos) and 12 (4 embryos) sections were analyzed for control and Neurog2, respectively. ***p < 0.001 (Student t test). (C, E) Left: Transverse sections of the dorsal NT in the Hes5-VNP transgenic line transfected at E2 with the indicated constructs harvested at E3 and immunostained for Venus (green). Transfection is reported by H2B-iRFP expression (red). Right: Quantification of the Hes5-VNP signal intensity in HuCD− cells in control (non-electroporated side), (C) Neurog2, and (E) ΔMaml1 conditions. A minimum of n = 84 cells (C) or n = 51 cells (E) collected from four embryos were analyzed for each group. ns, p > 0.05; ***p < 0.001 (Mann-Whitney U test). (D, F) Left: Transverse sections of the NT transfected at E2 with the indicated constructs, harvested 24 hae or 48 hae and immunostained for HuCD (red) to label neurons. Transfection is reported by GFP expression. Right: Quantification of the differentiation rate (number of HuCD+ cells on total transfected cells) 24 hae and 48 hae. Data represent mean + SEM. (D) For 24 hae, n = 13 (9 [file pbio.2004162.s003.tif]

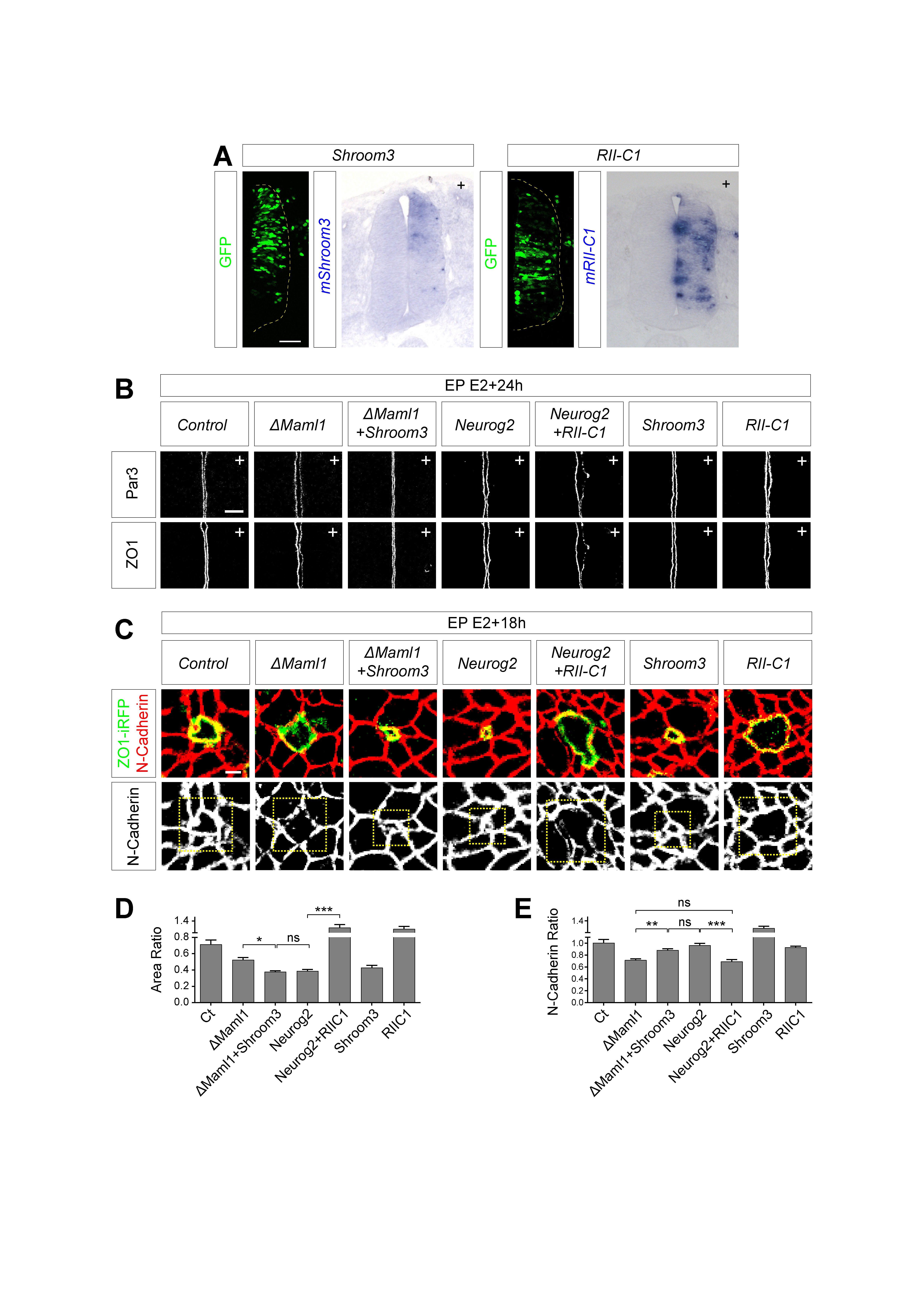

Supplement: S4 Fig — (A) Transverse sections of the NT transfected at E2 with the indicated constructs and harvested at E3. Adjacent sections were used to visualize electroporation efficiency with GFP expression and to reveal mShroom3 or mRII-C1 expression by in situ hybridization. Scale bar represents 50 μm. (B) Transverse views of the NT transfected at E2 with the indicated constructs, harvested at E3 and immunostained for the apical markers Par3 and ZO1. + indicates the transfected side of the NT. Scale bar represents 25 μm. (C) Apical views of the NT at E2 transfected with ZO1-iRFP (green) along with the indicated constructs, harvested 18 hae and immunostained for N-Cadherin. The boxed areas indicate the cell of interest. Scale bar represents 2 μm. (D, E) Quantification of the apical area ratio (ratio of the area of a transfected cell on the mean area of four of its close non-transfected neighbors) and N-Cadherin level ratio (ratio of the average pixel intensity within the apical circumference of one transfected cell corrected by the background versus the mean of average pixel intensity of four of its close non-transfected neighbors). Data represent mean + SEM. ns, p > 0.05; *p < 0.05; **p < 0.01; ***p < 0.001 (one-way ANOVA). Underlying data are provided in S1 Data. E, embryonic day; GFP, green fluorescent protein; hae, hour after electroporation; iRFP, infrared fluorescent protein; ns, nonsignificant; NT, neural tube; Par3, Partition defective protein 3; RII-C1, Shroom3 binding site on ROCK2; Shroom3, shroom family member 3; ZO1, zonula occludens 1. (TIF) [file pbio.2004162.s004.tif]

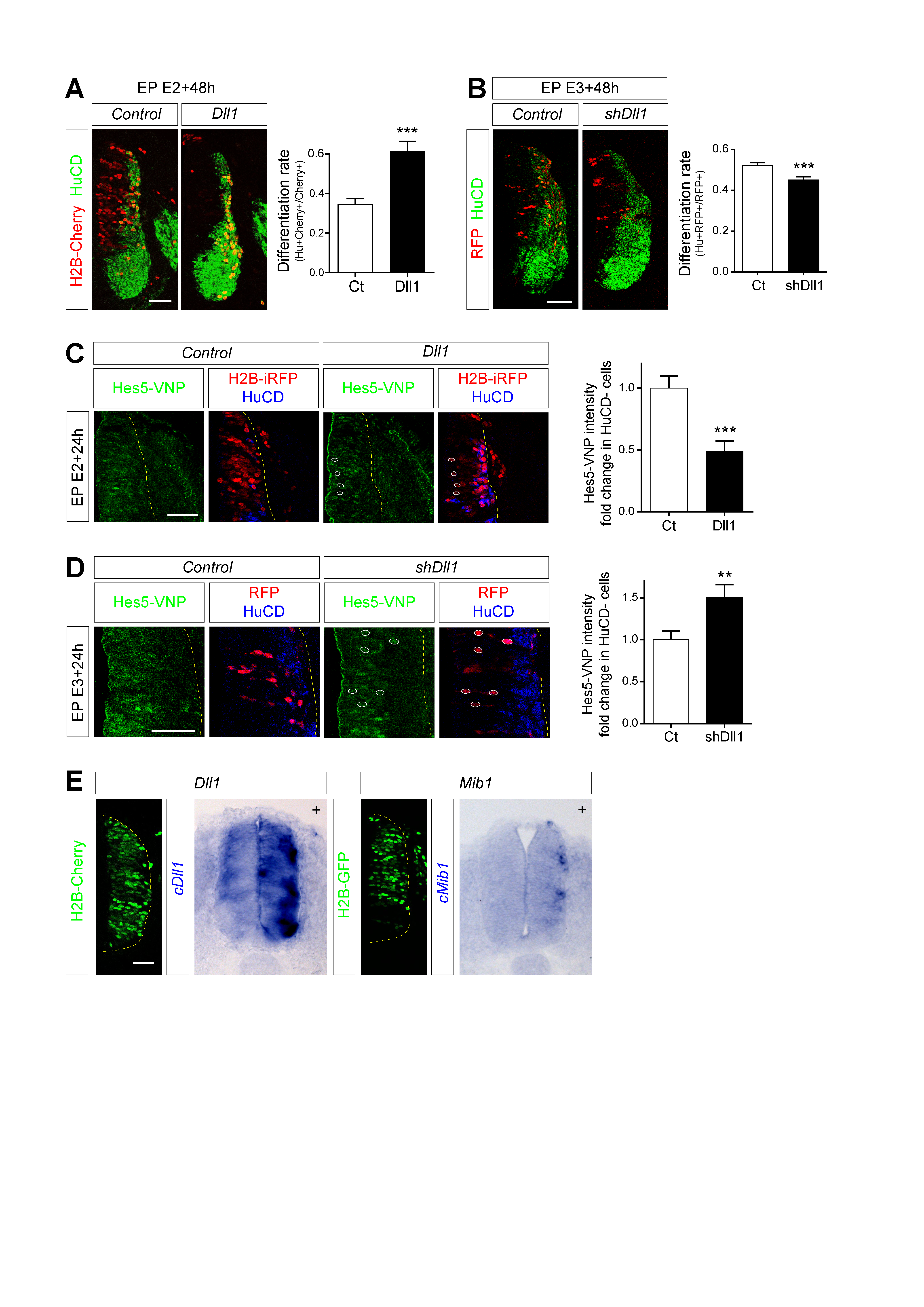

Supplement: S5 Fig — (A, B) Left: Transverse sections of the NT transfected at E2 (A) or E3 (B) with the indicated constructs, harvested at E4 (A) or E5 (B) and immunostained for HuCD (green) to label neurons. Transfection is reported by H2B-Cherry or RFP expression. In (B), electroporation was performed at low voltage (15 V) to obtain mosaic transfections. Right: Quantification of the differentiation rate (number of HuCD+ cells on total transfected cells). Data represent mean + SEM. (A) n = 12 and 15 sections collected from six embryos for each experimental group were analyzed for control and Dll1, respectively. (B) n = 36 sections (6 embryos) and 40 sections (8 embryos) were analyzed for control and shDll1, respectively. ***p < 0.001 (Student t test). (C, D) Left: Transverse section of the NT of the Hes5-VNP transgenic line transfected at E2 with Dll1 (C) or at E3 with shDll1 (D) constructs and their respective controls, harvested 24 hae and immunostained for Venus (green) and HuCD (blue) to label neurons. Transfection is reported by H2B-iRFP (C) or RFP (D) expression (red). Right: Quantification of Hes5-VNP intensity in HuCD− cells transfected with the indicated constructs at E2 (C) or E3 (D) with a normal (C) or low voltage (D) condition and harvested 24 hae. Data represent fold change compared to control. (C) n = 58 and 59 cells collected from six embryos for each experimental group were analyzed for control and Dll1, respectively. (D) n = 35 and 42 cells collected from 11 embryos for each experimental group were analyzed for control and shDll1, respectively. **p < 0.01; ***p < 0.001 (Mann-Whitney U test). Underlying data are provided in S1 Data. (E) Transverse sections of the NT transfected at E2 with the indicated constructs and harvested at E3. Adjacent sections were used to visualize electroporation efficiency with H2B-GFP or H2B-Cherry expression and to reveal cDll1 or cMib1 expression by in situ hybridization. + indicates the electroporated side of the NT. Scale bar represent [file pbio.2004162.s005.tif]

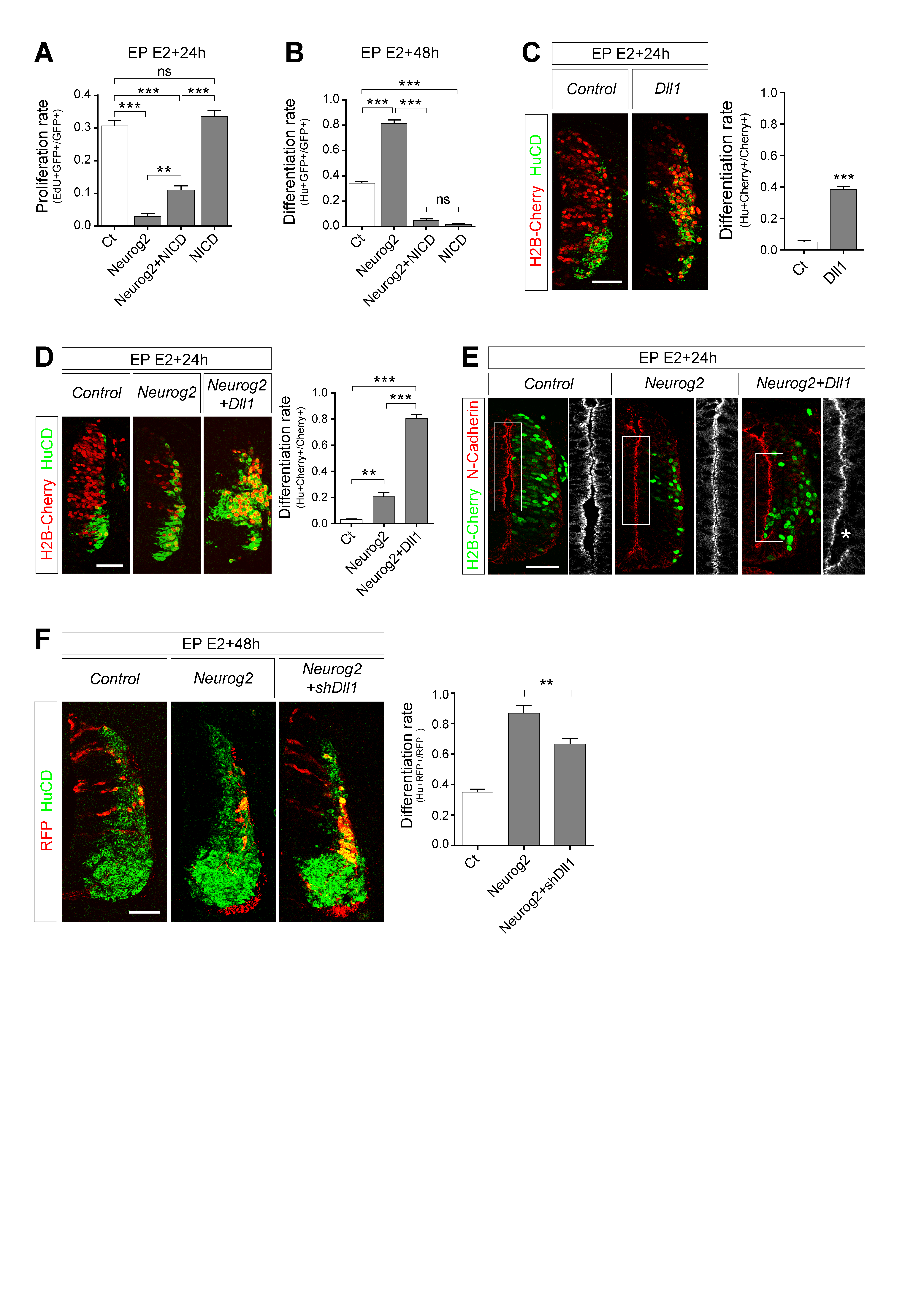

Supplement: S6 Fig — (A) Quantification of the proliferation rate (number of EdU+ cells on total transfected cells) 24 hae. Data represent mean + SEM. n = 10, 12, 23, and 20 sections collected from four embryos for each experimental group were analyzed for control, Neurog2, Neurog2+NICD, and NICD, respectively. (B) Quantification of the differentiation rate (number of HuCD+ cells on total transfected cells) 48 hae. Data represent mean + SEM. n = 9, 9, 9, and 7 sections collected from three embryos for each experimental group were analyzed for control, Neurog2, Neurog2+NICD, and NICD, respectively. ns, p > 0.05; **p < 0.01; ***p < 0.001 (one-way ANOVA). (C, D) Left: Transverse sections of the NT transfected at E2 with the indicated constructs, harvested at E3 and immunostained for HuCD (green) to label neurons. Transfection is reported by H2B-Cherry expression (red). Right: Quantification of the differentiation rate (number of HuCD+ cells on total transfected cells). Data represent mean + SEM. (C) n = 10 and 12 sections collected from four embryos for each experimental group were analyzed for control and Dll1, respectively. (D) n = 8, 9, and 15 sections collected from six embryos for each experimental group were analyzed for control, Neurog2, and Neurog2+Dll1, respectively. **p < 0.01; ***p < 0.001 (one-way ANOVA). (E) Transverse sections of the NT transfected at E2 with the indicated constructs, harvested at E3, and immunostained for N-Cadherin (red). Transfection is reported by H2B-Cherry expression (green). N-cadherin is down-regulated on the electroporated side upon double Neurog2+Dll1 expression; asterisk indicates breach to the ventricular wall. (F) Left: Transverse sections of the NT transfected at E2 with the indicated constructs, harvested at E4 and immunostained for HuCD (green) to label neurons. Transfection is reported by RFP expression (red). Right: Quantification of the differentiation rate (number of HuCD+ cells on total transfected cells). Data represent mean + SEM. **p < [file pbio.2004162.s006.tif]
